# A comprehensive study of arthropod and onychophoran Fox gene expression patterns

**DOI:** 10.1371/journal.pone.0270790

**Published:** 2022-07-08

**Authors:** Ralf Janssen, Christoph Schomburg, Nikola-Michael Prpic, Graham E. Budd

**Affiliations:** 1 Department of Earth Sciences, Palaeobiology, Uppsala University, Uppsala, Sweden; 2 AG Zoologie mit dem Schwerpunkt Molekulare Entwicklungsbiologie, Institut für Allgemeine Zoologie und Entwicklungsbiologie, Justus-Liebig-Universität Gießen, Gießen, Germany; 3 Fachgebiet Botanik, Institut für Biologie, Universität Kassel, Kassel, Germany; Laboratoire de Biologie du Développement de Villefranche-sur-Mer, FRANCE

## Abstract

Fox genes represent an evolutionary old class of transcription factor encoding genes that evolved in the last common ancestor of fungi and animals. They represent key-components of multiple gene regulatory networks (GRNs) that are essential for embryonic development. Most of our knowledge about the function of Fox genes comes from vertebrate research, and for arthropods the only comprehensive gene expression analysis is that of the fly *Drosophila melanogaster*. For other arthropods, only selected Fox genes have been investigated. In this study, we provide the first comprehensive gene expression analysis of arthropod Fox genes including representative species of all main groups of arthropods, Pancrustacea, Myriapoda and Chelicerata. We also provide the first comprehensive analysis of Fox gene expression in an onychophoran species. Our data show that many of the Fox genes likely retained their function during panarthropod evolution highlighting their importance in development. Comparison with published data from other groups of animals shows that this high degree of evolutionary conservation often dates back beyond the last common ancestor of Panarthropoda.

## Introduction

Fox gene transcription factors are characterized by the presence of an approximately 100 amino acid long DNA-binding motif, the so-called forkhead domain [1]. This domain forms three α-helices, three β-sheets and two wing-shaped structures. Fox genes are involved in various developmental processes and have been studied in a large number of animals including vertebrates [2–4], cephalochordates [5, 6], hemichordates [7], echinoderms [8], annelids [9], molluscs [9], cnidarians [10, 11] and even sponges [12, 13]. Among the Ecdysozoa, however, comprehensive studies are restricted to the dipteran fly *Drosophila melanogaster* [14, 15] and the nematode worm *Caenorhabditis elegans* [16] (and references therein) (see Table 1 for *Drosophila* gene names). Data from other groups of ecdysozoans and other arthropods are relatively sparse and often only address single Fox genes [e.g. 17–22].

**Table 1:** *Drosophila* synonyms.

	Commonly used *Drosophila* names	*Drosophila* synonyms
*FoxA*	*forkhead (fkh)*	*CG10002*
*FoxB*	*fd96Ca* / *fd96Cb*	*fd4 (CG11921)* / *fd5 (CG11922)*
*FoxC*	*crocodile (croc)*	*fd1 (CG5069)*
*FoxD*	*fd59A*	*fd3 (CG3668)*
*FoxF*	*biniou (bin)*	*CG18647*
*FoxG*	*sloppy paired1 (slp1)* / *sloppy paired2 (slp2)* / *fd19B*	*CG16738* / *CG2939* / *CG9571*
*FoxJ1*	*FoxJ1*	*CG32006*
*FoxK*	*fd68A*	*CG11799*
*FoxL1*	*fd64A*	*fd2 (CG1132)*
*FoxN14*	*jumeau (jumu)*	*CG4029*
*FoxN23*	*checkpoint suppressor homologue (ches-1-like)*	*CG12690*
*FoxO*	*foxo*	*fd88A (CG3143)*
*FoxP*	*fd85E*	*CG16899*
*FoxQ2*	*fd102C*	*CG11152*
*FoxT*	*fd3F / Circadianly regulated gene 1* (*Crg-1*)	*CG12632* / *CG32788*

The first unified nomenclature for Fox genes was established by [23], defining 15 classes of Fox genes. In the following years, additional classes have been identified and four classes, FoxJ, FoxL, FoxN, and FoxQ each were subdivided into two (e.g. FoxJ into FoxJ1 and FoxJ2) [24]. Two of these, FoxR and FoxS are believed to represent vertebrate specific groups [25, 26]. Recently, yet another class of Fox genes, FoxT, has been identified that appears to be panarthropod specific [27, 28].

In this study, we analyzed the embryonic expression patterns of Fox genes in three arthropod species, representing main branches of Arthropoda, the red flour beetle *Tribolium castaneum*, the pill millipede *Glomeris marginata*, the common house spider *Parasteatoda tepidariorum*, and as a representative of Onychophora, the blue velvet worm *Euperipatoides kanangrensis*. Together, these species cover most of Panarthropoda. *Tribolium* serves as a representative of Hexapoda, that in contrast to *Drosophila* shows a more ancestral mode of development (e.g. [29, 30]). *Glomeris*, as a representative of Myriapoda, represents the sister group to hexapods + crustaceans (Pancrustacea) with which they form the Mandibulata (Myriapoda + Pancrustacea). *Parasteatoda* (as a representative of Chelicerata) represents the sister group to Mandibulata, and *Euperipatoides* (as a representative of Onychophora) likely represents the closest related outgroup to Arthropoda (e.g. [31, 32]).

**Fig 1:**
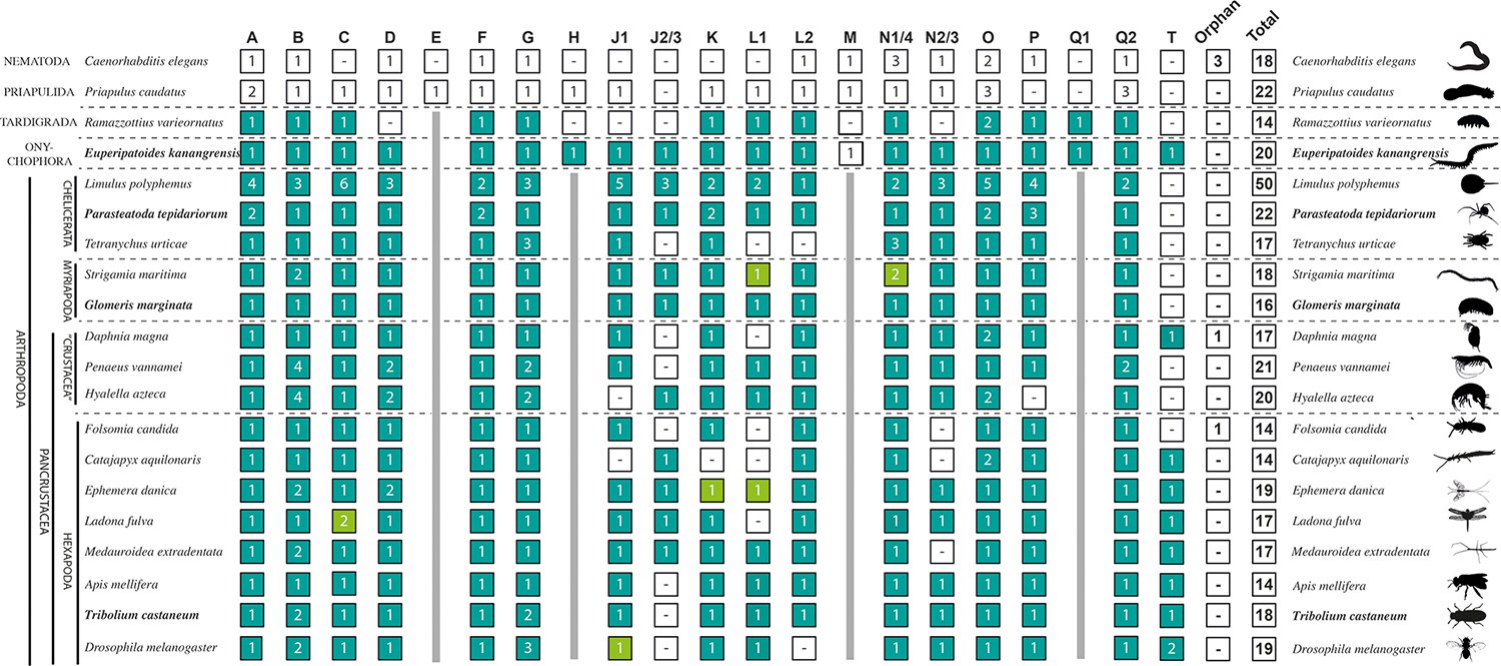
The complement of ecdysozoan Fox genes. Modified after [28] (their Fig 2). Green and light green boxes indicate that the gene falls in a given class of Fox genes in all or two of three analyses conducted in [28], respectively. Roman numbers in boxes indicate numbers of paralogs. A dash (-) indicates a predicted loss of a given lineage-specific Fox class gene. Solid vertical grey bars suggest losses of a class of Fox genes. Data on *Apis mellifera* stem partially from [198]. Note that the lack of *FoxJ23* in holometabolous insects in this overview does not represent a general loss in this group of animals (as it is present and some species), and that a possible *FoxQ1* gene was identified in a scorpion, albeit with weaker support [28]. A recent report by [62] listed 21 *Tribolium* Fox genes, but note that this is because three and two identical sequences of *FoxO* and *FoxP* respectively were incorporated in their list [62] (their Fig 2). The same analysis lists two *Drosophila FoxP* genes which appears to be a typo in their figure.

We analyzed the embryonic expression patterns of all identified Fox genes in these species (Fig 1). Whenever appropriate we also provide additional expression data on previously investigated Fox gene expression patterns. Expression data that simply add to or verify comprehensive earlier studies are provided in the supplementary data. In cases where a given Fox gene expression pattern has previously been investigated exhaustively, we refer to the published literature. Additionally, we compare the currently available data on Fox gene expression and function and try to recapitulate their potential roles during panarthropod evolution.

## Methods

### Animal husbandry and embryo preparation

Embryos were treated as described in [33] (*Tribolium*), [34] (*Glomeris*), [35] (*Parasteatoda*), and [36] (*Euperipatoides*). Developmental stages are defined as per [37] (*Tribolium*), [34] (*Glomeris*), [38] (*Parasteatoda*), and [39] (*Euperipatoides*).

### Sequence analysis

The phylogenetic relationship of all Fox genes identified in our research organisms has recently been investigated in [28]. For an overview of the Fox gene complements of arthropods and onychophorans, see Fig 1 (and S1 Fig).

### Gene cloning

For all species, RNA isolation of a mix of embryos representing different developmental stages, and subsequent cDNA synthesis were carried out as described in [34]. All gene fragments were amplified using gene specific primers (S1 Table) based on published genomes and transcriptomes, and Topo-TA cloned into the pCRII vector (Invitrogen, Carlsbad, CA, USA). Sequences were checked on an ABI3730XL analyser using Big Dye dye-terminators by a commercial sequencing service (Macrogen, Korea). Sequences identifiers of all investigated panarthropod Fox genes are listed in S2 Table.

### Whole-mount *in-situ* hybridization and DNA staining

All whole-mount *in-situ* hybridizations were performed as described in [40]. Cell nuclei were detected using 4-6-Diamidin-2-phenylindol (DAPI). Incubation in 2 μg/ml DAPI in phosphate buffered saline with 0.1% Tween-20 (PBST) for 30 minutes was followed by extensive washes in PBST to remove excess DAPI.

### Data documentation

Embryos were photographed using a Leica DC490 digital camera equipped with a UV light source mounted onto a MZ-FLIII Leica dissection microscope. Brightness, contrast, and color values were adjusted in all images using the image processing software Adobe Photoshop CC 2018 (for Apple Macintosh (Adobe Systems Inc. San Jose, CA, USA).

## Results

### Gene expression patterns

#### FoxA

Tribolium FoxA is first expressed in the yolk (not shown), and at later stages in the primordia of the stomodaeum and the proctodaeum (S2A Fig). Additional expression appears along the ventral midline in segmental clusters (S2A–S2C Fig, slim arrow) and laterally in the head lobes (S2A–S2C Fig, short arrow). At later developmental stages, after germ band retraction, it is expressed in the brain and in the hindgut, including the Malpighian tubules (S2 Fig). Some aspects of FoxA expression in Tribolium have been reported previously by [41].

*Glomeris FoxA* is first expressed in the primordia of the hindgut and the foregut. When the proctodaeum and the stomodaeum form, *FoxA* expressing cells sink in and form the through gut (S3A–S3D Fig). At late developmental stages, additional expression appears in the ventral nervous system (VNS) (S3D Fig, arrow). For further descriptions of *Glomeris FoxA* expression, see also [42].

First expression of *Parasteatoda FoxA-1* (described as *FoxA* in [43]) appears at stage 3 in the center of the germ disc, and at stage 4 at its rim (S4A and S4B Fig). At stage 5, single cells spread from the center of the disc and scatter over the disc and the dorsal field (S4C–S4E Fig). These cells are likely endodermal and may contribute to the developing gut as they are expressed in very similar patterns as the endodermal marker genes *serpent* and *hepatocyte nuclear factor 4* [44], (see also [42]). Later, *FoxA-1* is expressed in broad segmental patches along the ventral midline, that in subsequent stages form a continues domain along the midline (S4F and S5 Figs). At stage 10 and subsequent stages, most of the expression of *FoxA-1* disappears and transcripts only remain in the head and in the most posterior segments (S5G–S5I Fig).

**Fig 2:**
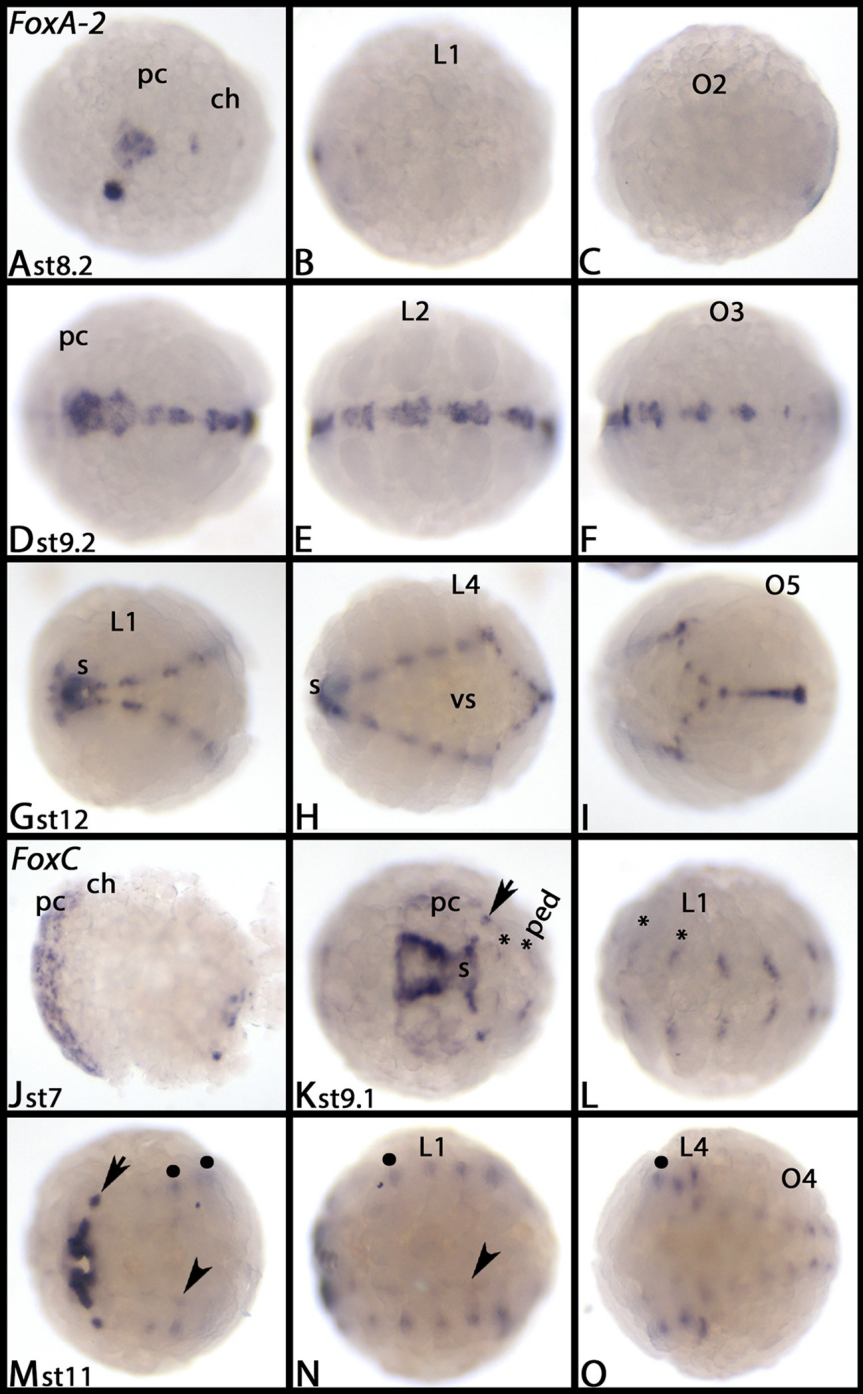
Expression of *Parasteatoda FoxA-2* (A-I) and *FoxC* (J-O). In all panels, anterior is to the left, ventral view. Panels A, D, G, K, and M view of anterior with head. Panels B, E, H, L, and N view of middle part with walking limbs. Panels C, F, I, O view of opisthosoma. Arrows in panels K and M point to lateral expression in the head. The arrowheads in panels M and N point to faint expression at the ventral rim of the split germ band. Asterisks in panels K and L mark expression in the VNS. Filled circles in panels M-O mark expression at the base of the walking limbs. Abbreviations in Table 2. DAPI staining of the embryos shown in this figure are presented in S6 Fig.

*Parasteatoda FoxA-2* is expressed later and first transcripts are detectable at around stage 8 in the stomodaeum and in segmental patches along the midline in anterior segments (Fig 2A–2C). At stage 9, all segments express *FoxA-2* in the midline (Fig 2D–2F). In contrast to *FoxA-1*, expression of *FoxA-2* does not disappear from central segments, but instead persists throughout the investigated developmental stages (Fig 2G–2I). Unlike *Fox-A1*, *Fox-A2* is not expressed in the dorsal field (S4 Fig cf. panels F and G). S6 Fig shows DAPI staining of the embryos shown in Fig 2.

**Table 2:** Abbreviations.

(av)	anlage of the anal valves
(P)	anlage of the proctodaeum
(S)	anlage of the stomodaeum
(T1/pmx)	anlage of the first trunk segment and the postmaxillary segment
a	anus
A	abdominal segment
an	antenna (-bearing segment)
av	anal valves
br	Brain
ch	chelicera (-bearing segment)
dee	dorsal extraembryonic ectoderm
df	dorsal field
e	eye
ect	ectoderm
fap	frontal appendage (the onychophoran antenna)
h	heart
hl	head lobe
j	jaw
L	leg (-bearing segment)
lb	labium (-bearing segment)
ic	intercalary segment
lr	labrum
m	mouth
m-a	mouth-anus furrow
mes	mesoderm
mp	Malpighian tubules
mx	maxilla (-bearing segment)
md	mandible (-bearing segment)
O	opisthosomal segment
oc	ocular region
P	proctodaeum
ped	pedipalp (-bearing segment)
pl	pleuropodia
pp	posterior pit (blastoporal region)
pc	pre-cheliceral region
vns	ventral nervous system
S	stomodaeum
saz	segment addition zone
sp	slime papilla (-bearing segment)
st	developmental stage
t	tail
T	trunk segment
vs	ventral sulcus

*Euperipatoides FoxA* is first expressed in the mouth-anus (m-a) furrow and ventral tissue between the developing germ bands (S7A Fig). After closure of the m-a furrow, *FoxA* remains expressed in the mouth and the anus, as well as in tissue lining the ventral margins of the germ band proper. This expression persists throughout further development (S7B and S7C Fig). For further expression details, see also [18, 42].

#### FoxB

Expression of *FoxB* genes in the here investigated arthropods and the onychophoran have recently been described in detail in [20, 45]. In all species, FoxB genes are expressed in the ventral sector of all appendages, except for the labrum of arthropods where expression is dorsal, and the onychophoran frontal appendages that do not express *FoxB* (S8 Fig). Additionally, *FoxB* is expressed in the ventral nervous system in all species (S8B, S8C, S8E, S8F, S8J, S8L, S8N and S8P Fig slim arrows). Early during development, in *Tribolium* both FoxB paralogs are expressed ubiquitously (S8A and S8D Fig). In *Glomeris*, first expression appears in the anlagen of the anal valves (S8G and S8H Fig). In the spider, *FoxB* is also expressed around the mouth/stomodaeum (S8K Fig).

#### FoxC

*Tribolium FoxC* is first expressed in an anterior cap, which refines to expression in the mouth primordium (S9A and S9B Fig). When the germ band begins to elongate, *FoxC* is expressed ventrally in the head around the mouth (S9C Fig). This expression in principle remains throughout further development (S9D and S9F Fig). Additional expression appears in the proctodaeum, in the brain and in the VNS (the latter is marked by arrows in S9C–S9F Fig). After germ band retraction, *FoxC* is also expressed in the developing heart (cf. expression of *FoxF*, below) (S9F Fig, short arrow). Expression of *FoxC* in the head has also been reported previously by [46] Economou and Telford (2009).

At the blastoderm stage (stage 0), *Glomeris FoxC* expression is in the form of an anterior cap, very similar to the expression of other head-patterning genes in *Glomeris* [47] (S3E Fig). This domain transforms into expression surrounding the mouth and in the anterior head skeleton [cf. 48, 49] (S3F–S3H Fig). For the head patterning role of *Glomeris FoxC*, see also [47].

In *Parasteatoda* stage 7 embryos, expression of *FoxC* is in the anterior margin of the embryo (Fig 2J). Later, this domain refines into domains along either side of the mouth primordium, the pharynx and a pair of small dots (Fig 2K and 2M, arrow) in the pre-cheliceral region (Fig 2K and 2L). Segmental patches of expression are in the VNS (Fig 2K and 2L, asterisks), and at the base of the walking limbs (Fig 2M–2O, filled circles). Faint expression is at the ventral rim of the split germ band (Fig 2M and 2N, arrowheads). Some aspects of *FoxC* expression have also been described by [50]. S6 Fig shows DAPI staining of the embryos shown in Fig 2.

**Fig 3:**
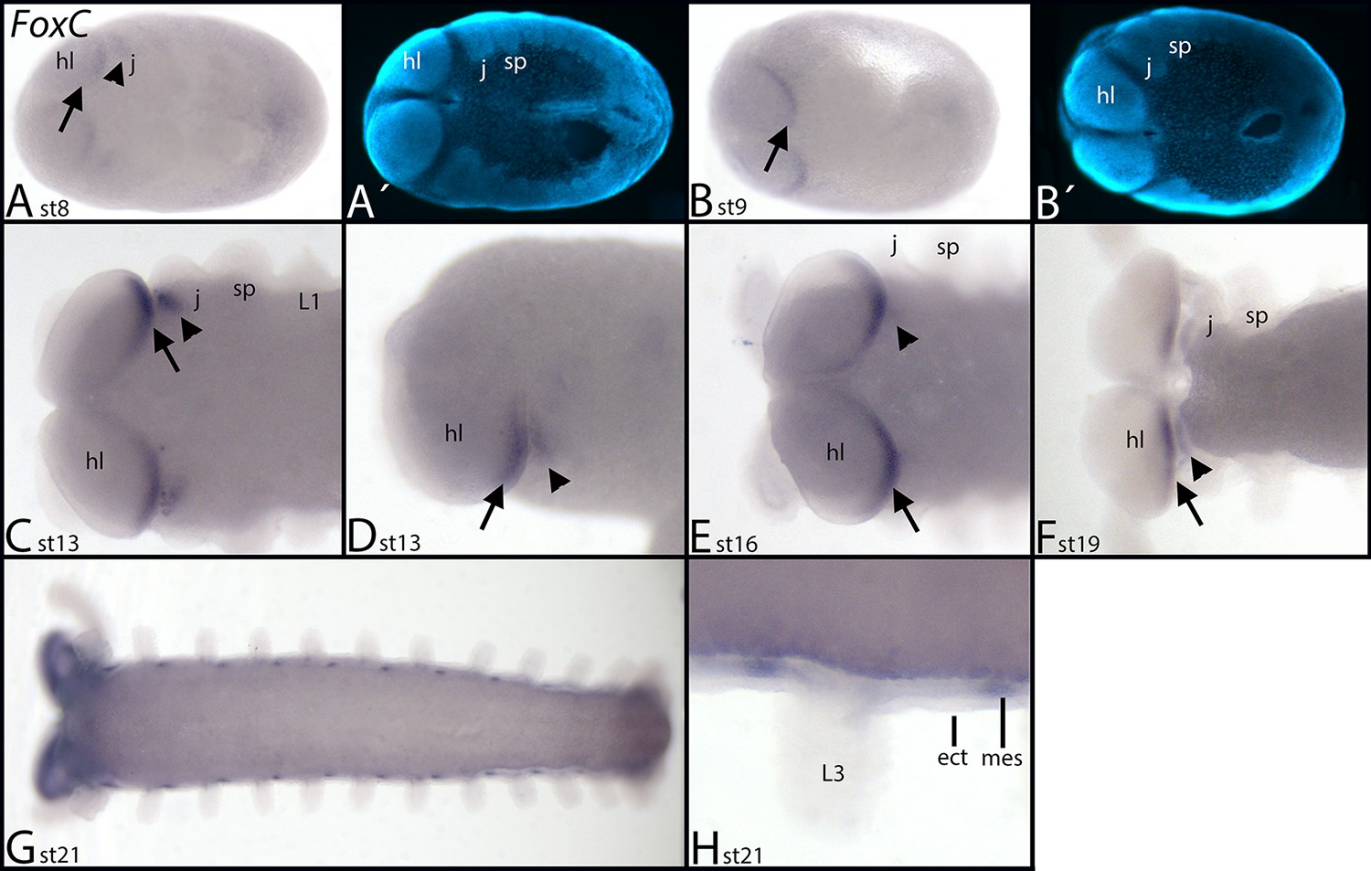
Expression of *Euperipatoides FoxC*. In all panels, anterior is to the left, ventral views, except panel D, lateral view, dorsal up. A´ and B´ represent DAPI staining of the embryos shown in A and B. In all panels, arrows point to expression at the posterior margin of the head lobes, and arrowheads indicate expression in the anterior of the jaw-bearing segment. Abbreviations in Table 2.

At stage 8, expression of *Euperipatoides FoxC* appears in the posterior of the head lobes and the anterior of the jaw-bearing segment (Fig 3A–3F, arrow and arrowhead respectively). This remains the only expression until stage 21 when mesodermal segmental patches appear along the anterior-posterior axis of the embryo (Fig 3G and 3H).

#### FoxD

**Fig 4:**
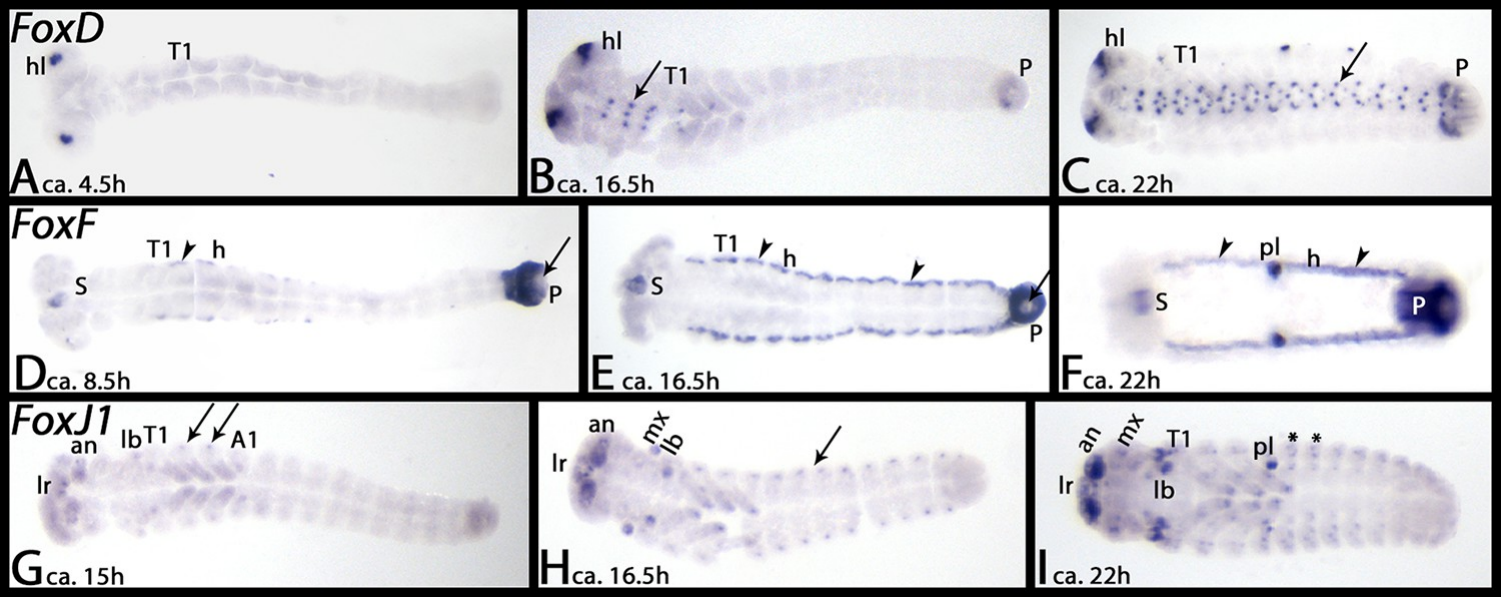
Expression of *Tribolium FoxD* (A-C), *FoxF* (D-F) and *FoxJ1* (G-I). In all panels, anterior is to the left, ventral view. Embryos are flat-mounted. Arrows in panels B and C mark expression in the VNS. Arrows in panels D and E point to the proctodaeum. Arrows in panels G and H mark dorsal expression. Arrowheads in panels D-F mark expression in the heart (dorsal tube). Asterisks in panel I mark dots of expression in dorsal tissue. Abbreviations in Table 2.

*Tribolium FoxD* appears in the form of two dots in the head lobes when germ band elongation has almost completed (Fig 4A). Later, segmental dots appear in an anterior to posterior progression in the VNS (arrows), and in the proctodaeum (Fig 4B and 4C).

**Fig 5:**
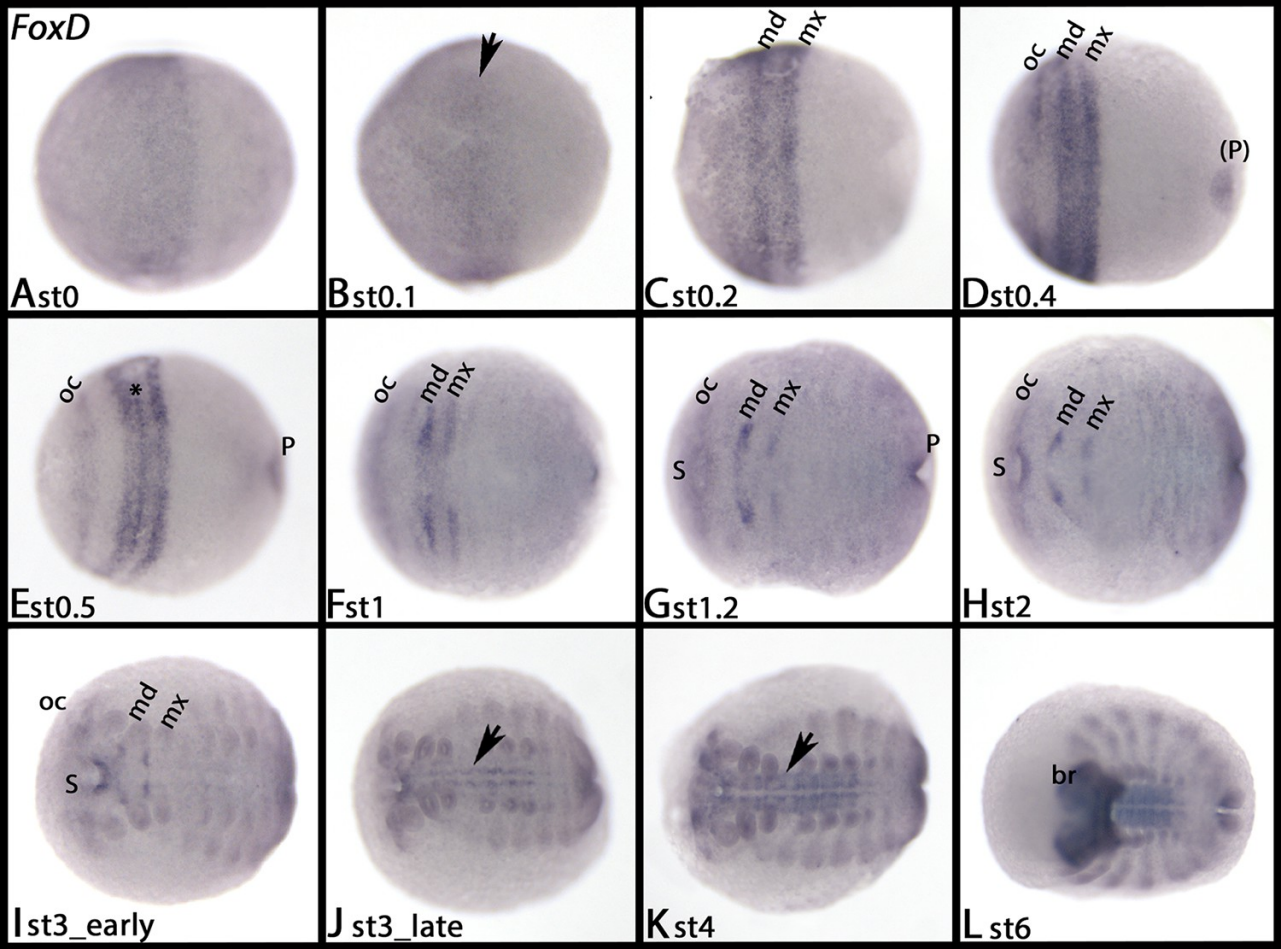
Expression of *Glomeris FoxD*. In all panels, anterior is to the left, ventral views. The arrow in panel B marks an appearing stripe of expression in the (likely) mandibular segment primordium. The asterisk in panel E marks the mandibular segment. Arrows in panels J and K mark expression in the VNS. Abbreviations in Table 2.

At stage 0, *Glomeris FoxD* is expressed in the form of an anterior cap (Fig 5A). Within this cap, a single stripe of enhanced expression appears; this stripe most probably represents expression in the primordium of the mandibular segment (Fig 5A, arrow). This assumption is based on the position of the stripe, the fact that we can follow the fate of the stripe over time, and the fact that the mandibular segment is often patterned first [51] (Fig 5B–5E). Shortly after formation of the first stripe, a second stripe appears at the posterior edge of the cap (Fig 5C). Then a third stripe appears that represents the ocular region (Fig 5D). At the same time, expression disappears from tissue between the ocular region and the mandibular stripe, and expression in the primordium of the proctodaeum appears (Fig 5D). Expression in the mandibular domain refines into a narrow but strong stripe (Fig 5E and 5F, asterisk in panel E). At stage 1.2, expression is still in the mandibular and the maxillary segment (Fig 5G and 5H). After this expression has disappeared, strong *de novo* expression appears in the VNS (Fig 5I–5K, arrows). At late developmental stages, *FoxD* is strongly expressed in the brain (Fig 5L). From stage 3 onwards, all tissue expresses *FoxD* weakly (Fig 5I–5L).

**Fig 6:**
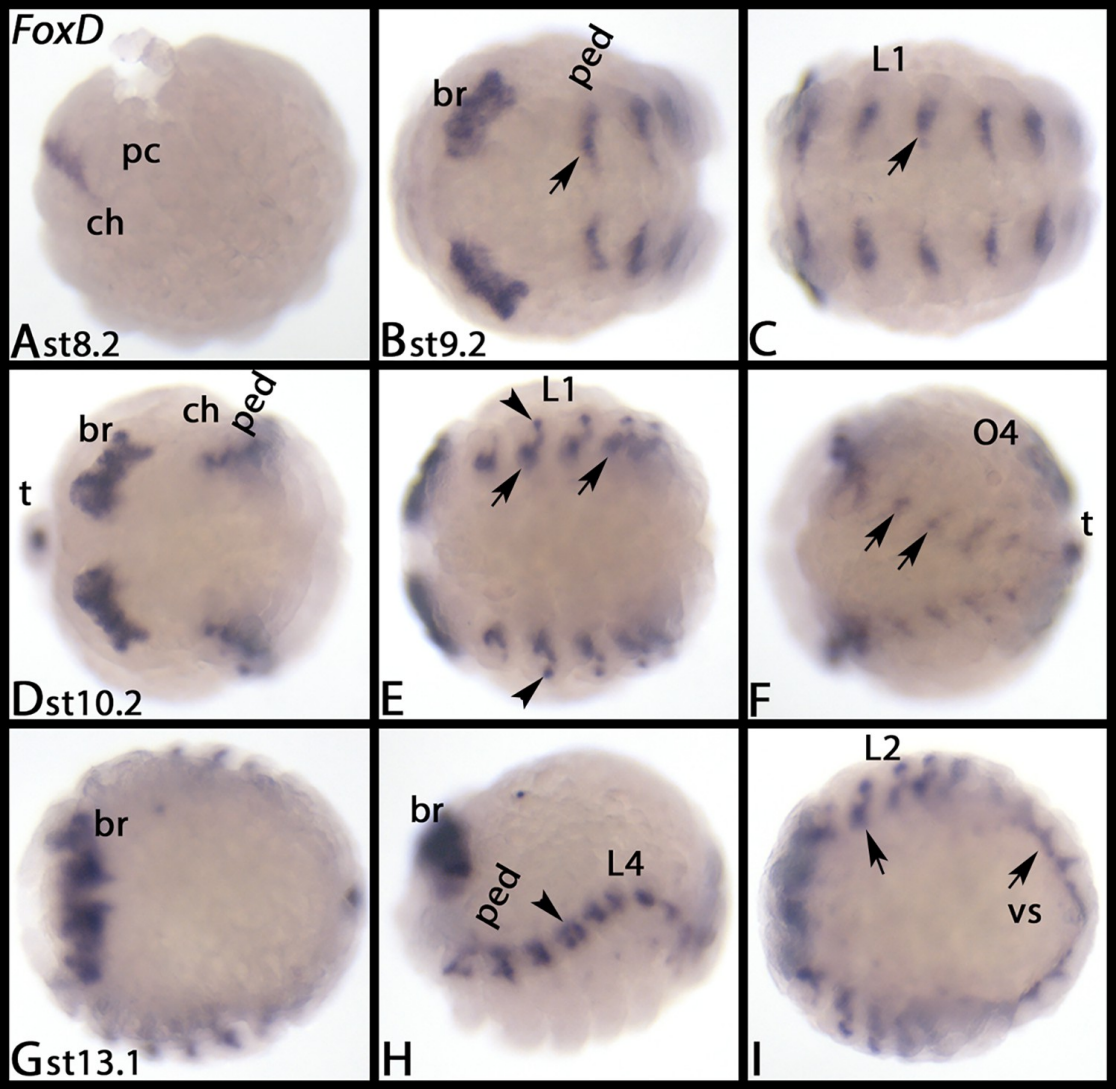
Expression of *Parasteatoda FoxD*. In all panels, anterior is to the left, ventral views, except panels G, dorsal view and A, H, lateral view. Each row represents the same embryo, except for first row. Arrows point to expression in the VNS. Arrowheads point to expression at the base of the limbs. Abbreviations in Table 2. DAPI staining of the embryos shown in this figure are presented in S10 Fig.

*Parasteatoda FoxD* is first expressed at sage 8.2 as a transverse stripe in the pre-cheliceral region (Fig 6A). Later, this domain splits into two domains in the developing brain (Fig 6B, 6D, 6G and 6H). At the same time, segmental patches of expression appear in the VNS of all segments except for the cheliceral segment (Fig 6B, 6C, 6E, 6F and 6I, arrows), and at the base of the walking limbs and pedipalps (Fig 6E and 6H, arrowheads). S10 Fig shows DAPI staining of the embryos shown in Fig 6.

**Fig 7:**
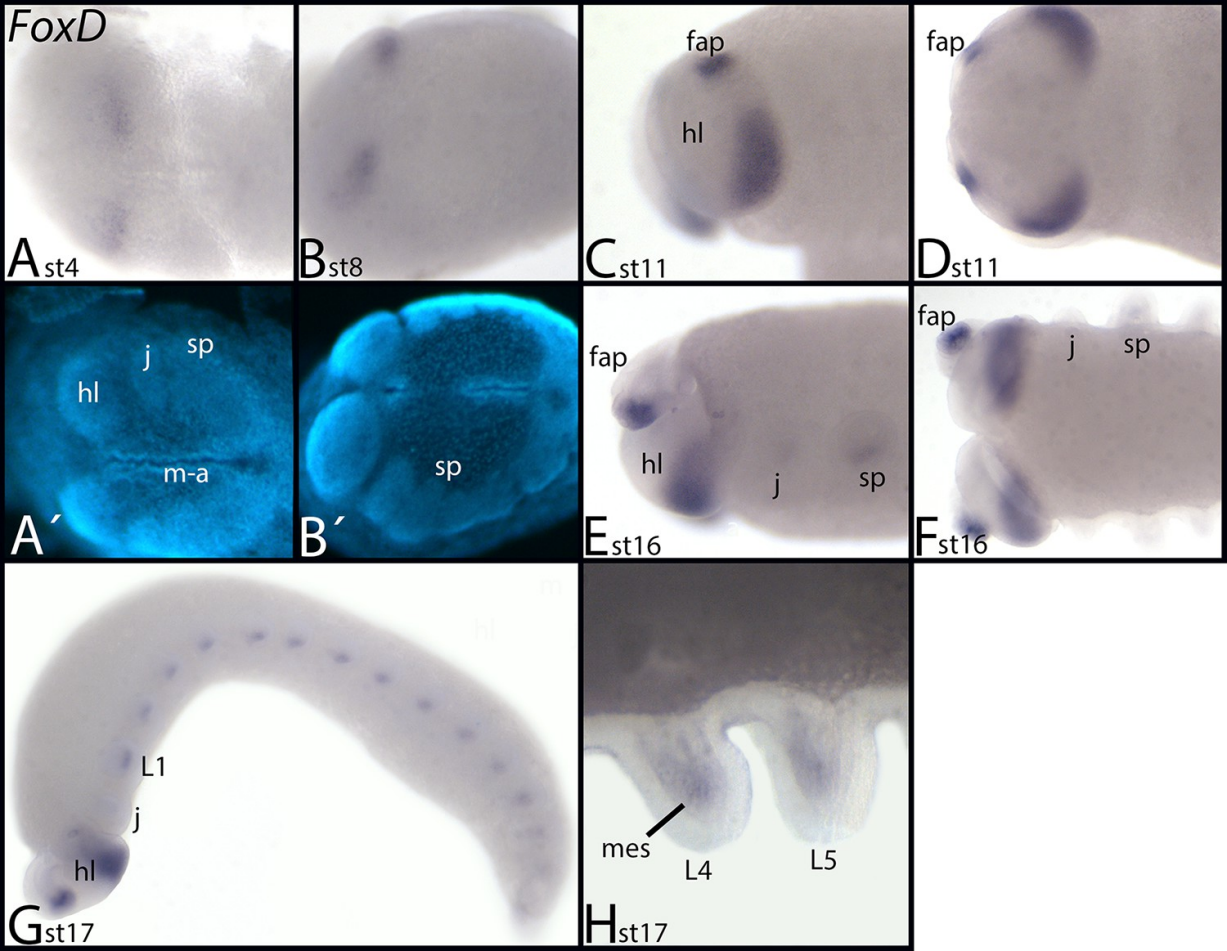
Expression of *Euperipatoides FoxD*. In all panels, anterior is to the left. Panels A, B, and F, ventral view; panels C, E and G, lateral view, panel D, view of the head. A´/B´ represent DAPI staining of the embryos shown in A/B. Abbreviations in Table 2.

*Euperipatoides FoxD* is first expressed in the posterior of the head lobes, but in a region slightly more anterior than that of *FoxC* (Fig 7A–7G). At stage 11, additional expression appears in the tips of the frontal appendages (Fig 7C–7G). At stage 16, weak mesodermal expression appears inside the jaws and the slime papillae (Fig 7E and 7F). At later stages, this expression is also present in the legs (Fig 7G and 7H).

#### FoxF

*Tribolium FoxF* is first expressed exclusively in the stomodaeum and the proctodaeum, although the most posterior tip of the embryo remains free from expression throughout development (not shown). Later, additional expression appears in the heart (Fig 4D–4F, arrowheads) (cf. expression of heart-patterning genes in [52]). Note the unspecific staining of the pleuropodia (pl) in panel F.

**Fig 8:**
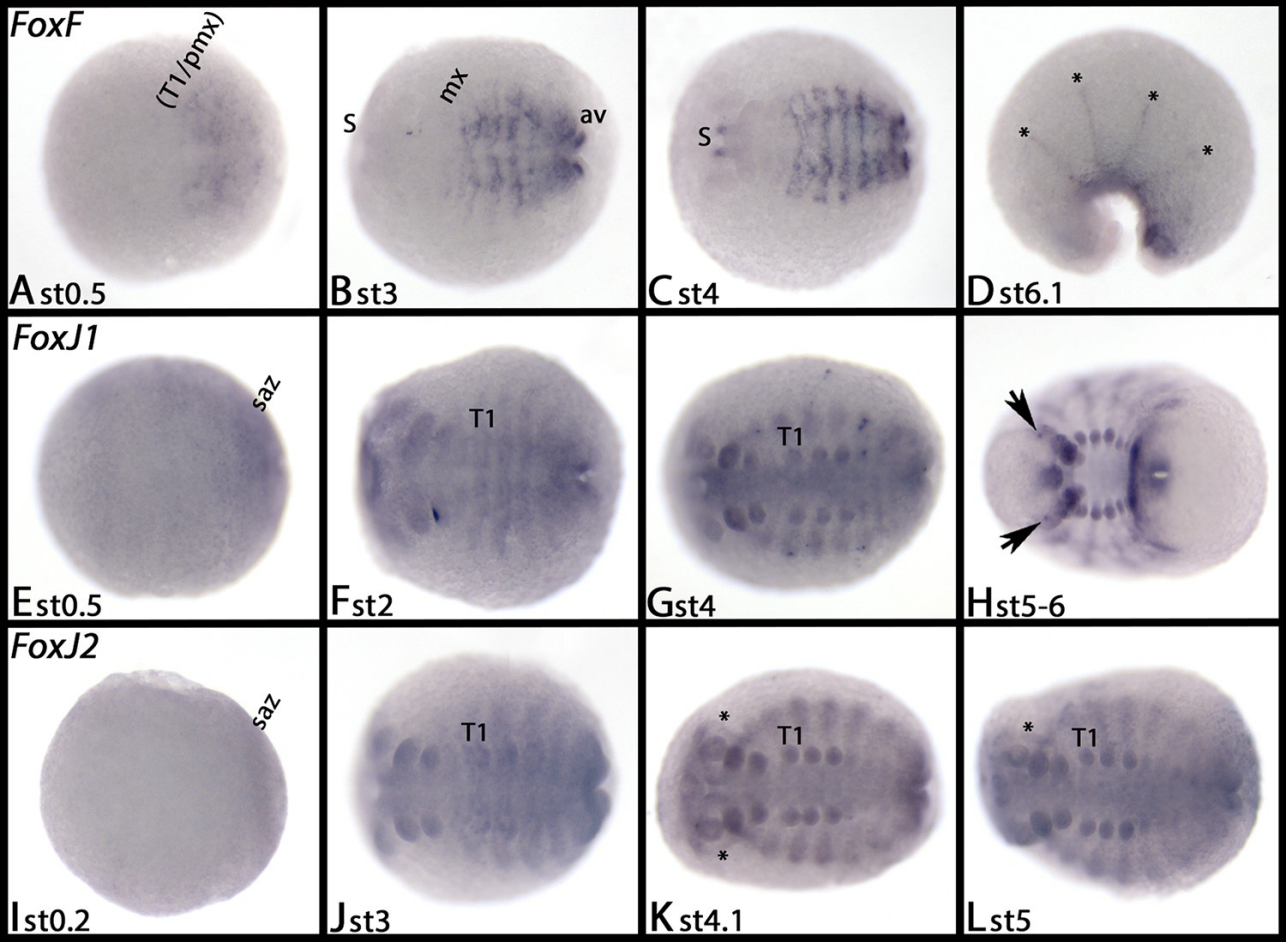
Expression of *Glomeris FoxF* (A-D), *FoxJ1* (E-H) and *FoxJ2* (I-L). In all panels, anterior is to the left, ventral views except panel D (lateral view, dorsal up). Asterisks in panel D mark expression in the tergite borders. Arrows in panel H mark dot-like expression in the head. Asterisks in panels K and L mark an area that is free of expression lateral in the head. Abbreviations in Table 2.

At stage 0.5, expression of *Glomeris FoxF* appears in a diffuse pattern in the trunk segments; the head remains free from expression (Fig 8A). At later developmental stages, this expression refines into segmental stripes that cover the middle of the ventral and dorsal segmental units of the trunk (Fig 8B–8D and S11A Fig). In late stage 3 embryos, a dot of expression appears on either side lateral to the mouth (Fig 5C and S11A Fig). Later, this tissue forms part of the foregut. Enhanced expression is inside the anal valves from stage 3 onwards (Fig 8B–8D and S11A and S11B Fig). At late developmental stages, *FoxF* is also expressed in the grooves between the developing tergites (Fig 8D, asterisks) [cf. 34, 53].

**Fig 9:**
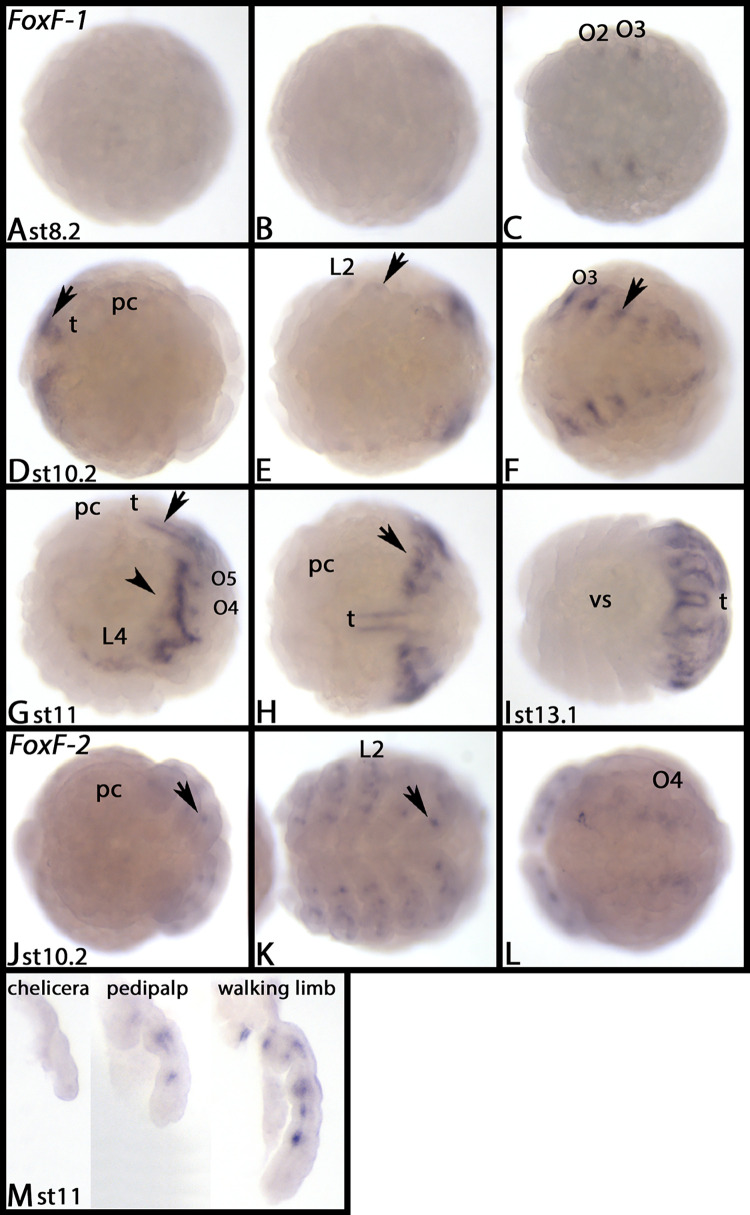
Expression of *Parasteatoda FoxF-1* (A-I) and *FoxF-2* (J-M). In all panels, anterior is to the left, ventral views, except panels G and M, lateral views, and H, dorsal view. Each row represents the same embryo, if not of different developmental stage. Arrows in panels D-H point to expression in the opisthosoma, and in panels J and K the legs. Abbreviations in Table 2. DAPI staining of the embryos shown in this figure are presented in S12 Fig.

*Parasteatoda FoxF-1* is first expressed at stage 8.2 in the form of faint patches in the second and third opisthosomal segments (Fig 9A–9C). Later, all opisthosomal segments express *FoxF-1*, mostly in dorsal tissue (Fig 9D–9I, arrows). Additional expression appears in the form of a faint stripe dorsal to the limbs of the prosoma and in the tail (Fig 9G and 9H). At late stages, almost the complete opisthosoma expresses *Fox-F1* (Fig 9I). *FoxF-2* is expressed in a subset of mesodermal cells in the pedipalps and the walking limbs in embryos of stage 10.2 (Fig 9J–9M, arrows) and later (not shown). S12 Fig shows DAPI staining of the embryos shown in Fig 9.

**Fig 10:**
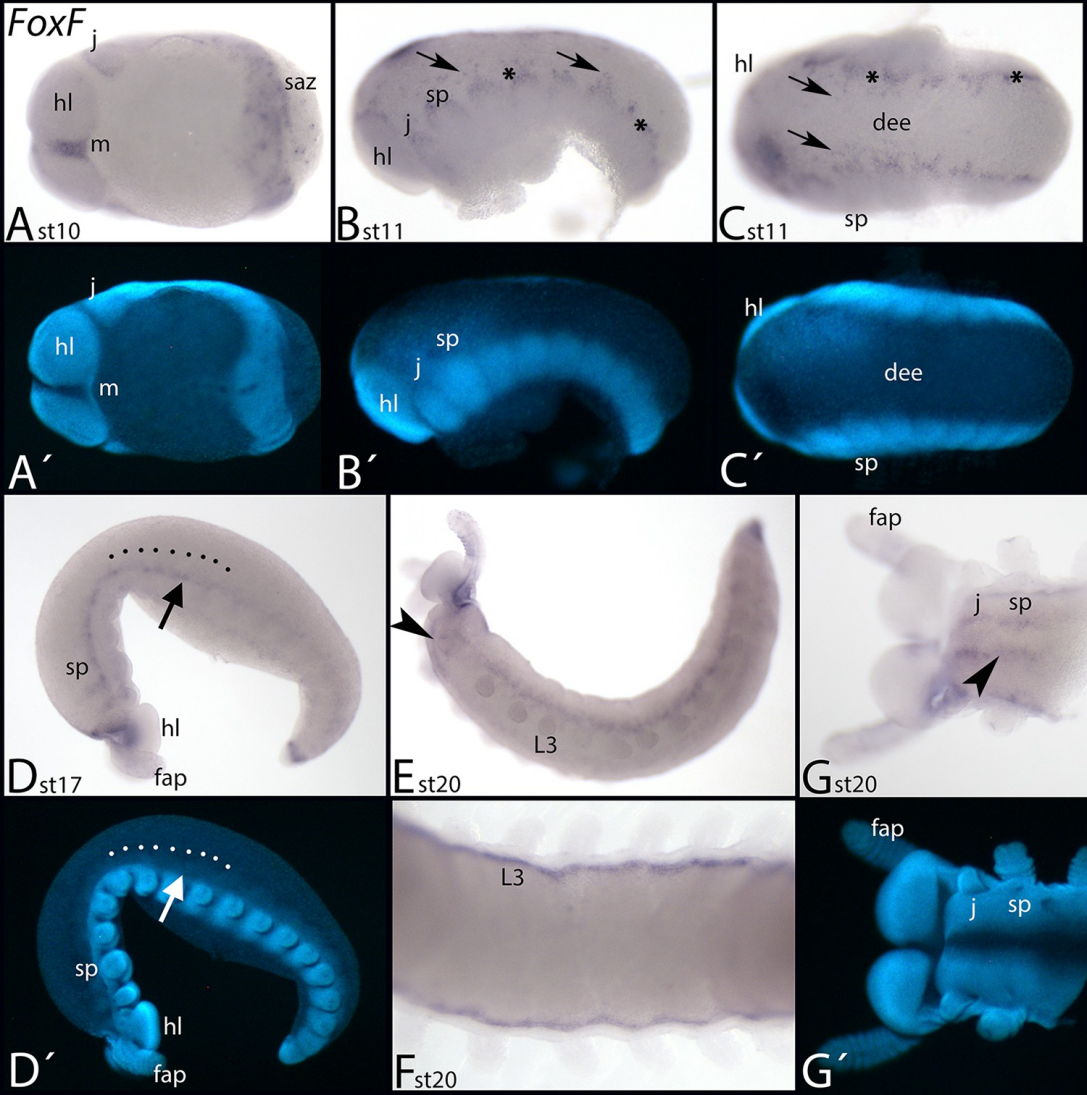
Expression of *Euperipatoides FoxF*. In all panels, anterior is to the left. Panels A and G, ventral view; panels B, D and E, lateral view; panels C and F, dorsal view. A´-D´ and G´ represent DAPI staining of the embryos shown in A-D and G. Arrow in panel D points to expression dorsally abutting the limb buds. Dashed line in panel D indicates dorsal margin of the germ band. Asterisks in panels B and C mark expression at the dorsal rim of the embryo; arrows in B and D point to expression in the dorsal extraembryonic tissue. Arrowheads in panels E and G mark expression in the ventral nervous system. Abbreviations in Table 2.

*Euperipatoides FoxF* is first expressed anterior to the mouth, and in the form of a sharp band demarcating the anterior edge of the jaw-bearing segment (Fig 10A). A salt-and-pepper like expression is in the SAZ and newly formed segments (Fig 10A). At subsequent stages, expression is restricted to the dorsal edge of all segments (Fig 10B and 10C, asterisks), and some cells in the so-called dorsal-extraembryonic tissue (Fig 10B and 10C, arrows). At later stages this expression is not located at the dorsal edge of the embryo but in a position more ventrally, just dorsal to the position of the outgrowing limbs (Fig 10D–10F, arrow in panel D). From stage 20 onwards, additional expression appears in an anterior to posterior order along the ventral edge of the germ band (Fig 10E, 10G, arrowheads).

#### FoxG

A detailed description of *Tribolium FoxG-1* (*slp*) and *FoxG-2* (*slp2*) has recently been published in [54]. The expression and function of *slp* has also been studied by [55].

In *Glomeris*, the appearance of segmental stripes in the post-blastoderm stage embryo is complex (S3I Fig). At later developmental stages, expression is in the brain (ocular region, oc), along the ventral midline, the limbs, in lateral segmental patches (arrows in panels K and L) and as transverse stripes in newly forming posterior segments (S3J–S3L Fig). The segmental expression pattern of *Glomeris FoxG* has also been described previously by [56, 57].

**Fig 11:**
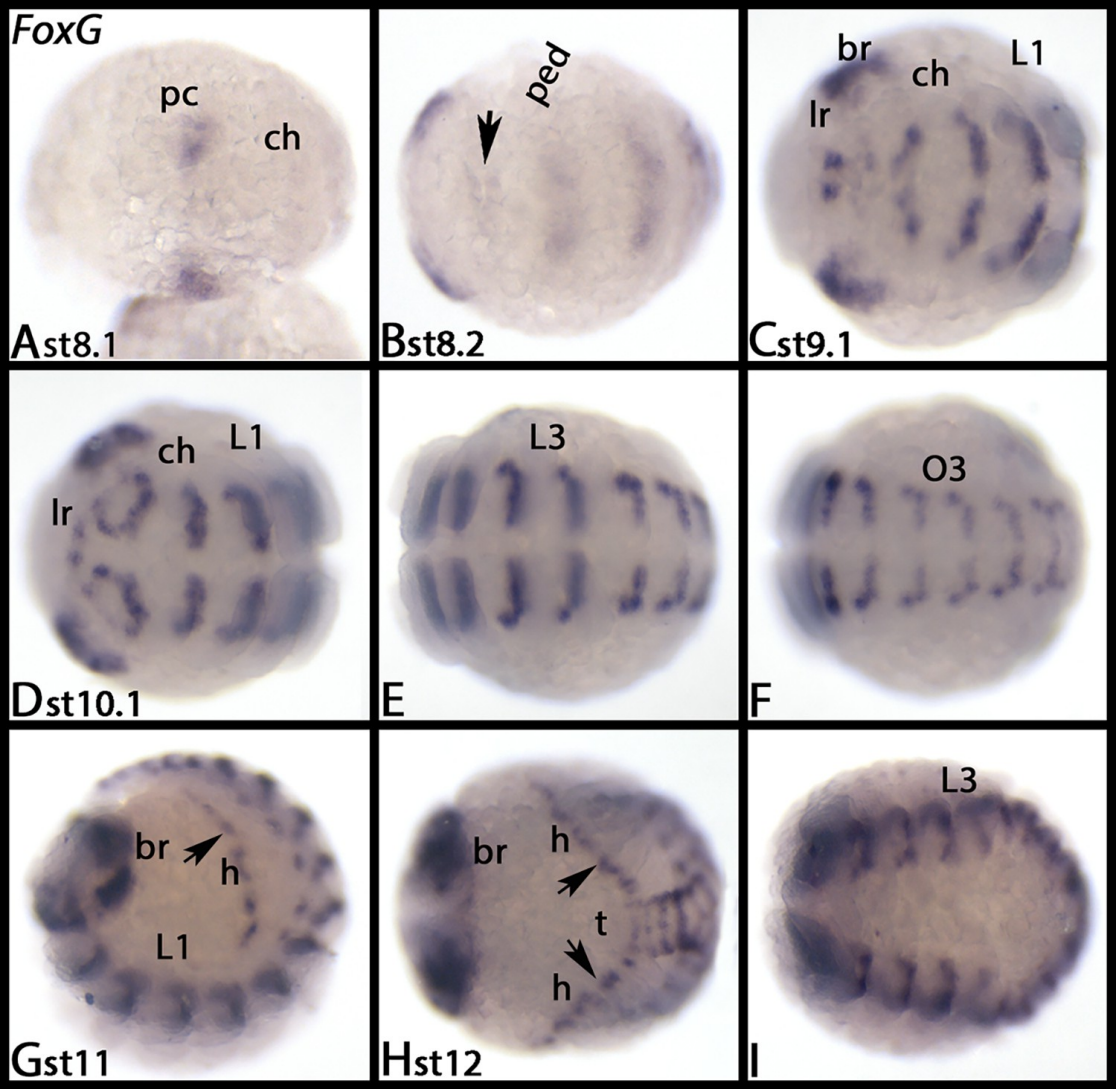
Expression of *Parasteatoda FoxG*. In all panels, anterior is to the left, ventral views, except panels G, lateral view and H, dorsal view. Each row (except first row, A-C) represents the same embryo. Arrow in panel B marks weak expression in the chelicerae segment. Arrows in panels G and H point to the heart. Abbreviations in Table 2. DAPI staining of the embryos shown in this figure are presented in S13 Fig.

*Parasteatoda FoxG* is first expressed in the pre-cheliceral region at stage 8.1 (Fig 11A), and shortly later transverse stripes of expression appear in all segments (Fig 11B and 11C). This segmental expression persists throughout development (Fig 11D–11I). Later, expression appears in the labral region (Fig 11D) and in the developing heart (Fig 11G and 11H, arrows). S13 Fig shows DAPI staining of the embryos shown in Fig 11.

Expression of *Euperipatoides FoxG* has been described by [39].

#### FoxH

**Fig 12:**
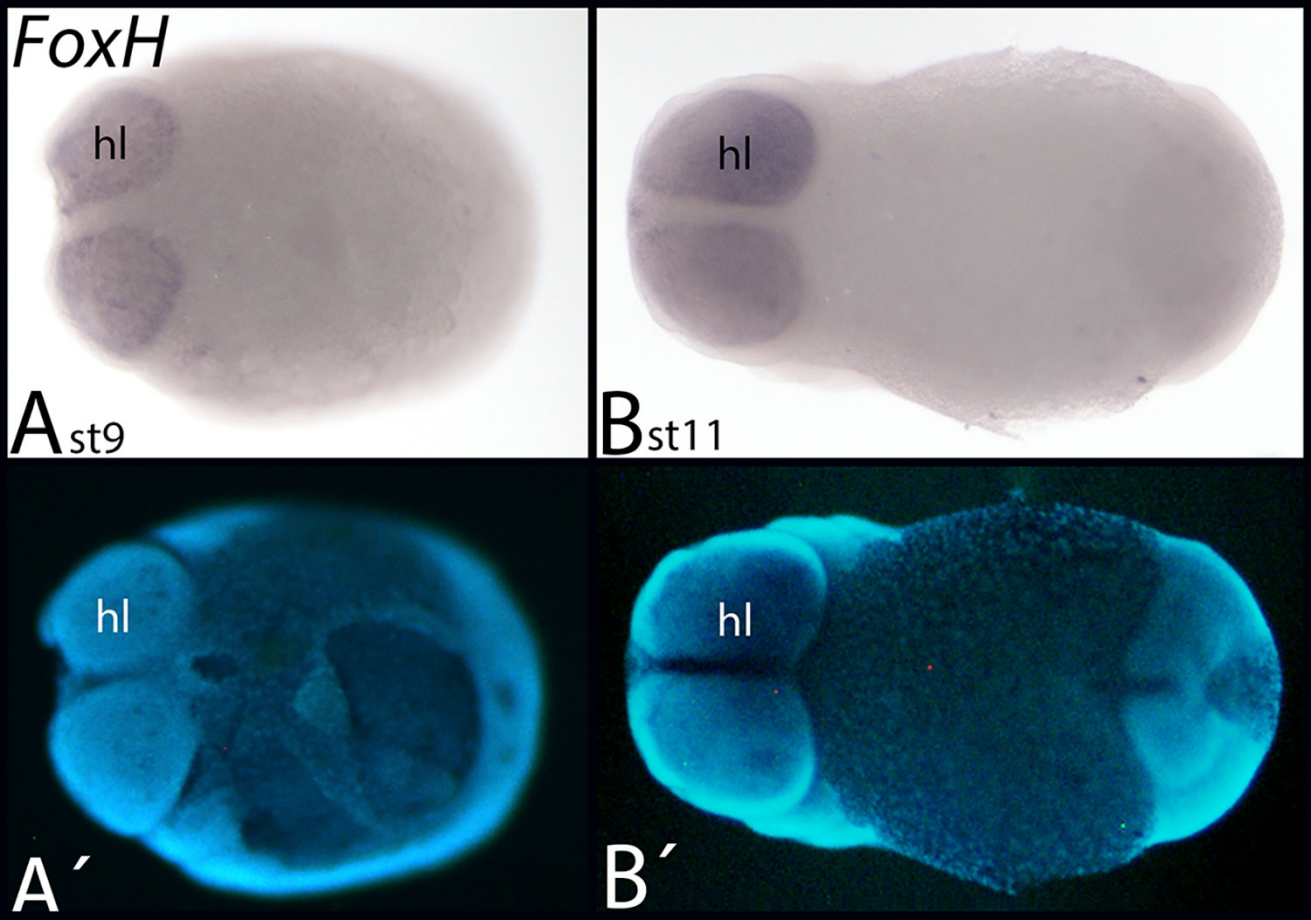
Expression of *Euperipatoides FoxH*. In all panels, anterior is to the left, ventral views. A´/B´ represent DAPI staining of the embryos shown in A/B Abbreviations in Table 2.

Among the investigated species, *FoxH* is only present in *Euperipatoides* where it is expressed inside the head lobes in early developmental stages (Fig 12).

#### FoxJ1

Expression of *Tribolium FoxJ1* appears by the end of germ band elongation in the form of two spots in the labrum, a diffuse pattern in the antennae and the walking limbs, a terminal domain in the labium, two spots in the first abdominal segment, and as defined spots dorsal in the second and third thoracic segment (arrows) (Fig 4G). Shortly after, additional dorsal expression appears in all abdominal segments (Fig 4H, arrow), the expression in the antennae becomes stronger, expression appears in the maxillae, and a spot of expression appears in the tip of the legs (Fig 4H). By the end of germ band retraction, the overall pattern is the same as described, with the exceptions that now several dorsal segmental spots are present (Fig 4I, asterisks), and that additional spots of expression appeared in the legs (Fig 4I).

*Glomeris FoxJ1* is expressed ubiquitously at all stages, but there is enhanced dot-like expression in the ocular region at late developmental stages (Fig 8E–8H and S14B Fig).

**Fig 13:**
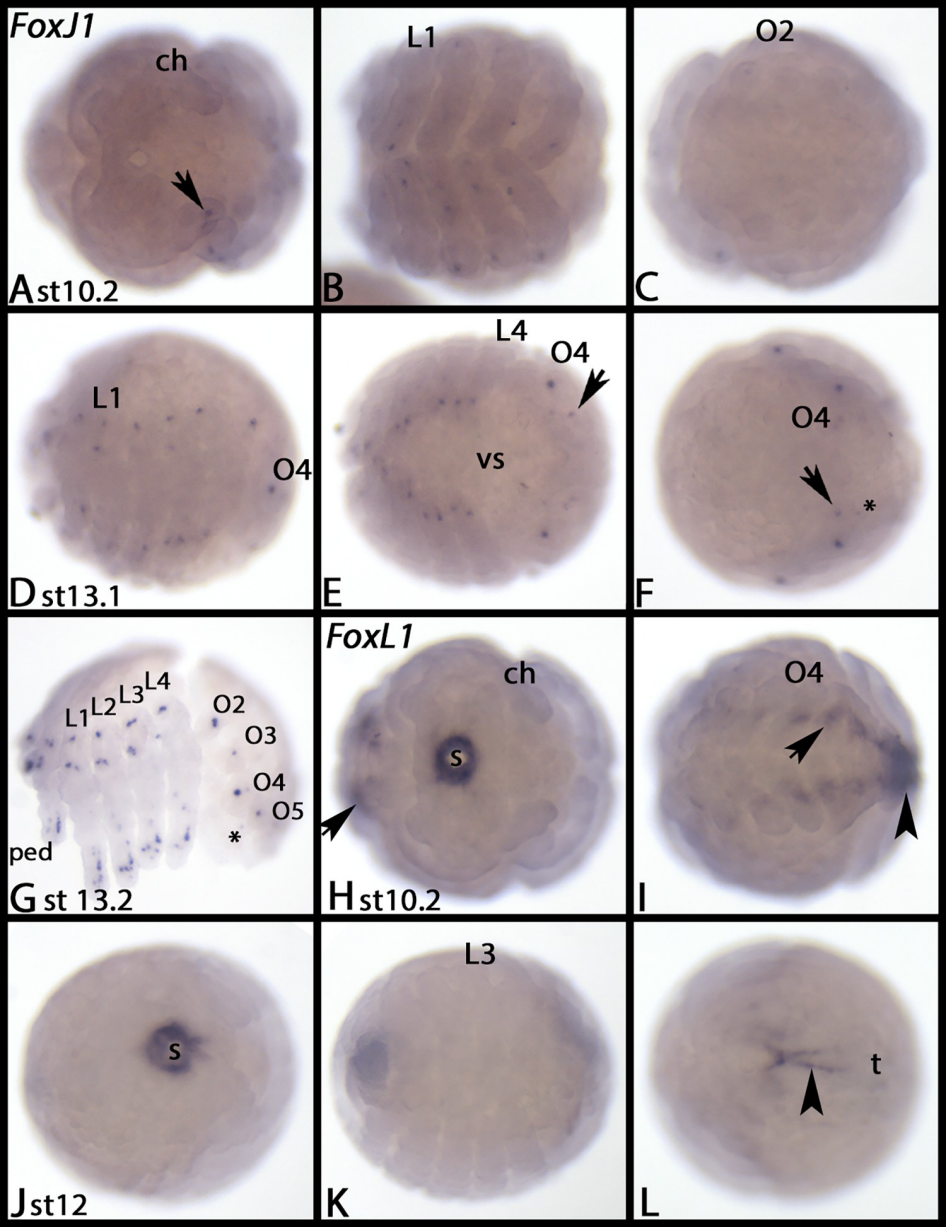
Expression of *Parasteatoda FoxJ1* (A-G) and FoxL1 (H-L). In all panels, anterior is to the left, ventral views, except panels D, lateral view, F, dorsal view and G, lateral view. Arrow in panel A marks expression in the chelicera. Arrows in panels E and F point to expression in the spinnerets on opisthosomal segment 5. Asterisks in panels F and G mark a second dot of expression in O5. Arrows in panels H and I point to expression ventral to the base of the limbs. Arrowheads in panels I and L point to expression in the tail region. Abbreviations in Table 2. DAPI staining of the embryos shown in this figure are presented in S15 Fig.

From stage 10.2 onwards, *Parasteatoda FoxJ1* is expressed in a number of single cells or clusters of cells in all limbs, including the labrum, spinnerets and book lungs (Fig 13A–13G). See figure legend for further information. S15 Fig shows DAPI staining of the embryos shown in Fig 13.

We did not detect expression of *Euperipatoides FoxJ1*.

#### FoxJ2

**Fig 14:**
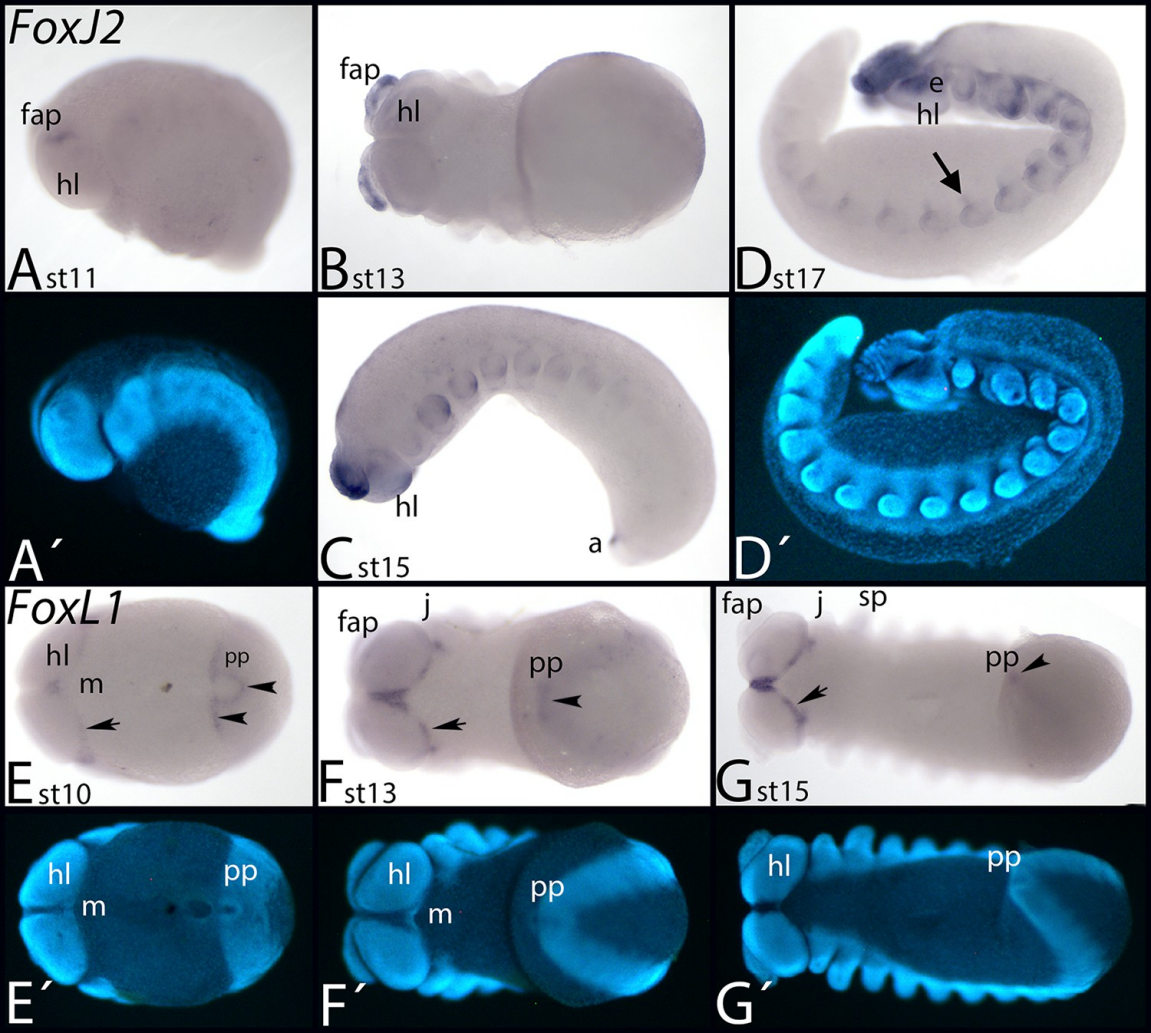
Expression of *Euperipatoides FoxJ2* (A-D) and *FoxL* (E-G). In all panels, anterior is to the left. Panels A, C and D lateral view. Panels B, and E-G ventral view. A´, D´ and E´-G´ represent DAPI staining of the embryos shown A, D and E-G. Arrow in panel D marks expression ventral to the base of the walking legs. Arrows and arrowheads in panels E-G point to expression at the posterior rim of the head lobes and the posterior pit respectively. Abbreviations in Table 2.

*Fox J2* is missing in *Tribolium*. *Glomeris FoxJ2* is expressed ubiquitously at all investigated developmental stages (Fig 8I–8L), except for late stages when transcripts are not seen in the lateral head region (Fig 8K and 8L, asterisks). *Parasteatoda FoxJ2* is either not expressed in the investigated developmental stages, or is expressed ubiquitously at a very low level (data not shown). *Euperipatoides FoxJ2* is first expressed in the frontal appendages (Fig 14A and 14B). Later, expression appears within the other appendages, in tissue dorsal to the limb buds, the anus (Fig 14C and 14D), and in a spot-like domain ventrally in the base of the limbs (Fig 14D, arrow).

#### FoxK

*FoxK* in *Tribolium*, *Glomeris*, *Euperipatoides*, *and FoxK-1* in *Parasteatoda* are expressed ubiquitously at all investigated developmental stages (data not shown). *Parasteatoda FoxK-2* either is not expressed in the investigated developmental stages, or is expressed ubiquitously at a very low level (data not shown).

#### FoxL1

**Fig 15:**
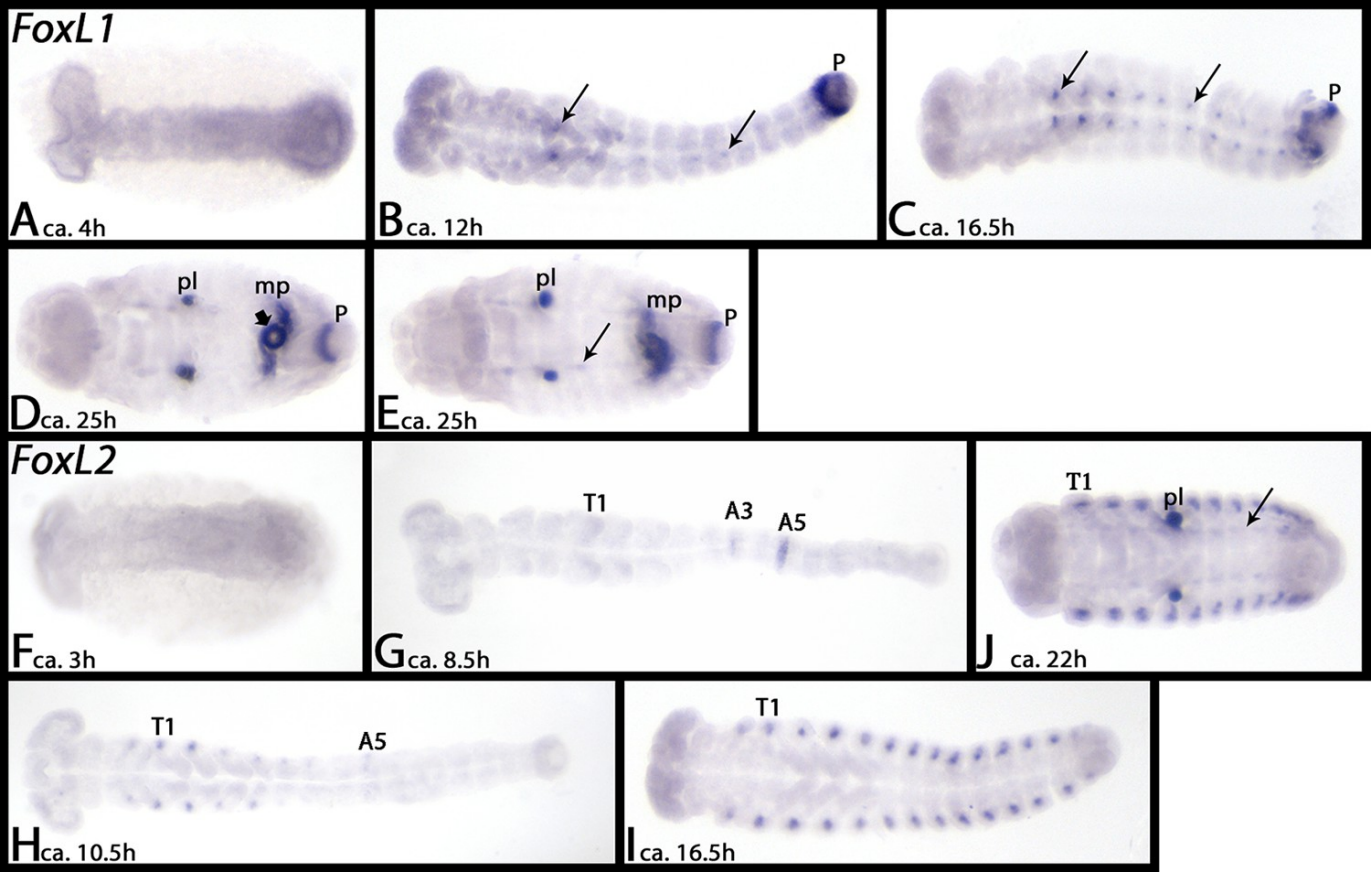
Expression of *Tribolium FoxL1* (A-E) and *FoxL2* (F-J). In all panels, anterior is to the left, ventral view. Embryos are flat-mounted, except embryos shown in panels A and F. Arrows in panels B, C, E and J point to expression in the VNS. Short arrow in panel D marks a ring of expression at the base of the Malpighian tubules. Note the unspecific staining of the pleuropodia in panels D, E, and J. Abbreviations in Table 2.

*Tribolium FoxL1* is first expressed ubiquitously (Fig 15A), but by the end of germ band elongation, expression appears in the proctodaeum and in the form of weak spots in the VNS of the thorax and the abdomen, but not the head (Fig 15B–15E, arrows). By the end of germ band retraction, the proctodeal domain has split into one in the anus and one encircling the end of the hindgut. By this stage, expression is also in the Malpighian tubules (Fig 15D and 15E).

**Fig 16:**
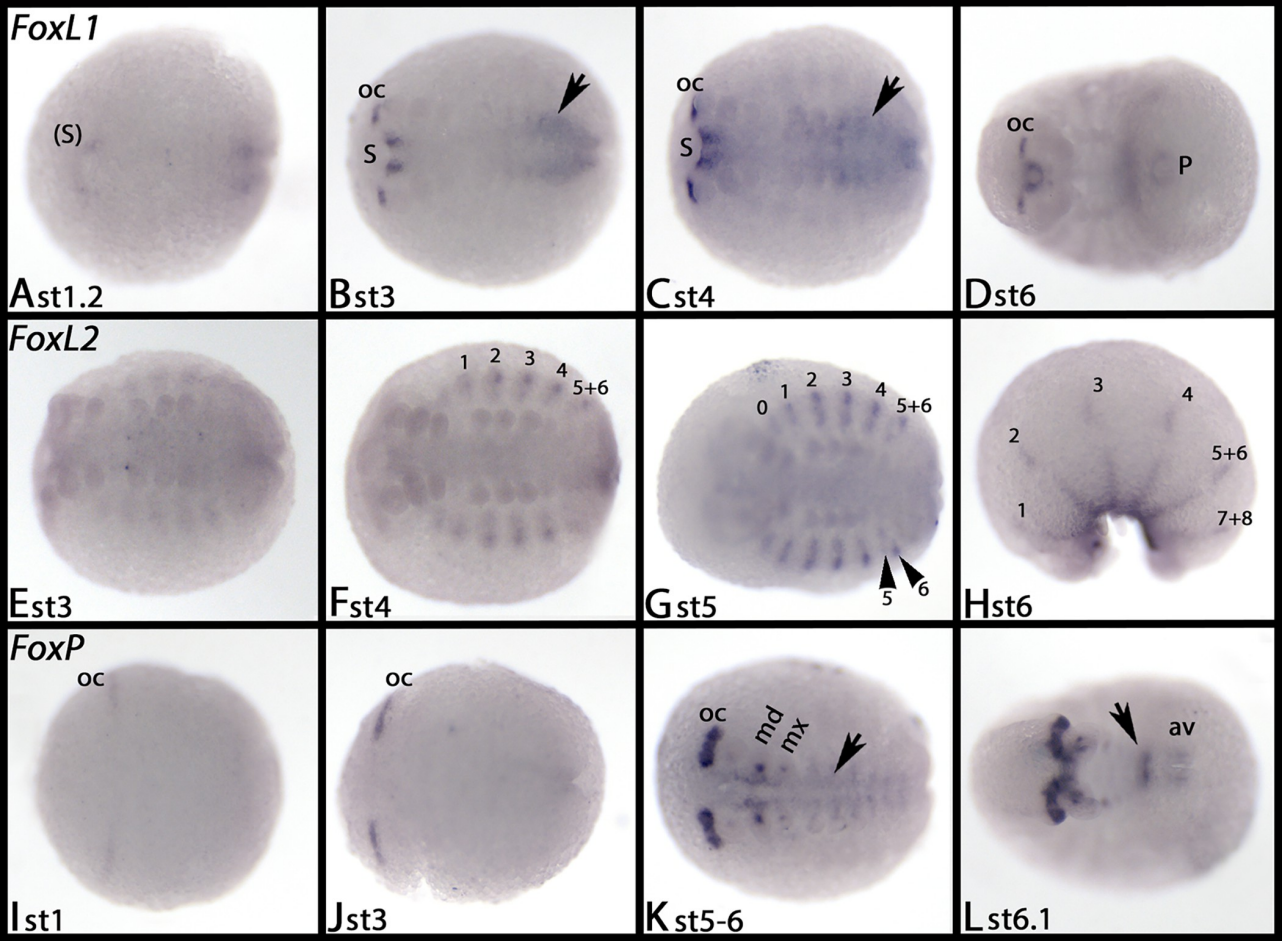
Expression of *Glomeris FoxL1* (A-D), *FoxL2* (E-H), and *FoxP* (I-L). In all panels, anterior is to the left, ventral views (except panel H, lateral view). Arrows in panels B and C point to expression in the developing midgut. Arrowheads in panel G point to two domains of expression in a fusing dorsal segmental unit. Roman numerals mark expression in the dorsal segmental units (cf. Janssen 2011). The arrow in panel K points to expression in the VNS. The arrow in panel L points to expression in the last formed segment. Abbreviations in Table 2.

*Glomeris FoxL1* is first expressed in a crescent-moon shaped domain anterior to the mouth primordium, and in the forming hindgut (Fig 16A). The anterior cells that express *FoxL1* then sink in and form part of the stomodaeum; *de novo* expression appears in the brain (Fig 16B) and persists throughout further development (Fig 16C). At stage 3, diffuse expression in the posterior half of the embryo appears that is likely associated with endodermal tissue of the developing gut (Fig 16B and 16C, arrows)

*Parasteatoda FoxL1* is expressed in the stomodaeum (Fig 13H, 13J and 13K), in tissue ventral to the opisthosomal limb buds (Fig 13H and 13I, arrows) and the tail region (Fig 13I and 13L, arrowheads). S15 Fig shows DAPI staining of the embryos shown in Fig 13.

*Euperipatoides FoxL1* is expressed in a small domain anterior to the mouth, the tissue posterior to the posterior edge of the head lobes (Fig 14E–14G, arrows), and in a horseshoe-like pattern in the posterior pit (the latter transforms into a simpler expression profile later during development) (Fig 14E–14G, arrowheads).

### FoxL2

At early developmental stages *Tribolium FoxL2* is either not expressed, or is expressed weakly and ubiquitously (Fig 15F). The first expression appears in the form of two transient segmental domains, one in the third abdominal segment, and one in the fifth (Fig 15G). By the end of germ band elongation, the abdominal expression domain in A3 has disappeared, and the one in A5 is very weak (Fig 15H). Segmental dots appear dorsal to the base of the labium, and the legs, and dorsally in the anterior abdominal segments (Fig 15H). Later, these dots are present in all abdominal segments (Fig 15I). Expression in the labial segment disappears at later developmental stages (Fig 15I and 15J). By the end of germ band retraction, weak expression in the VNS appears (Fig 15J, arrow).

*Glomeris FoxL2* is exclusively expressed in the mesoderm of the dorsal segmental units of the trunk (Fig 16E–16H, indicated by Roman numerals). This expression is comparable with that of the myogenic marker *nautilus* (*nau*), although *nau* is expressed earlier than *FoxL2* [cf. 58].

We isolated *Parasteatoda FoxL2* from maternal cDNA but we could not detect any expression during ontogenesis. We were unable to detect expression of *Euperipatoides FoxL2*.

### FoxM, FoxN14 and FoxN23

Expression of *FoxM*, *FoxN14* and *FoxN23* genes in the here investigated species has recently been described in [59].

### FoxO

**Fig 17:**
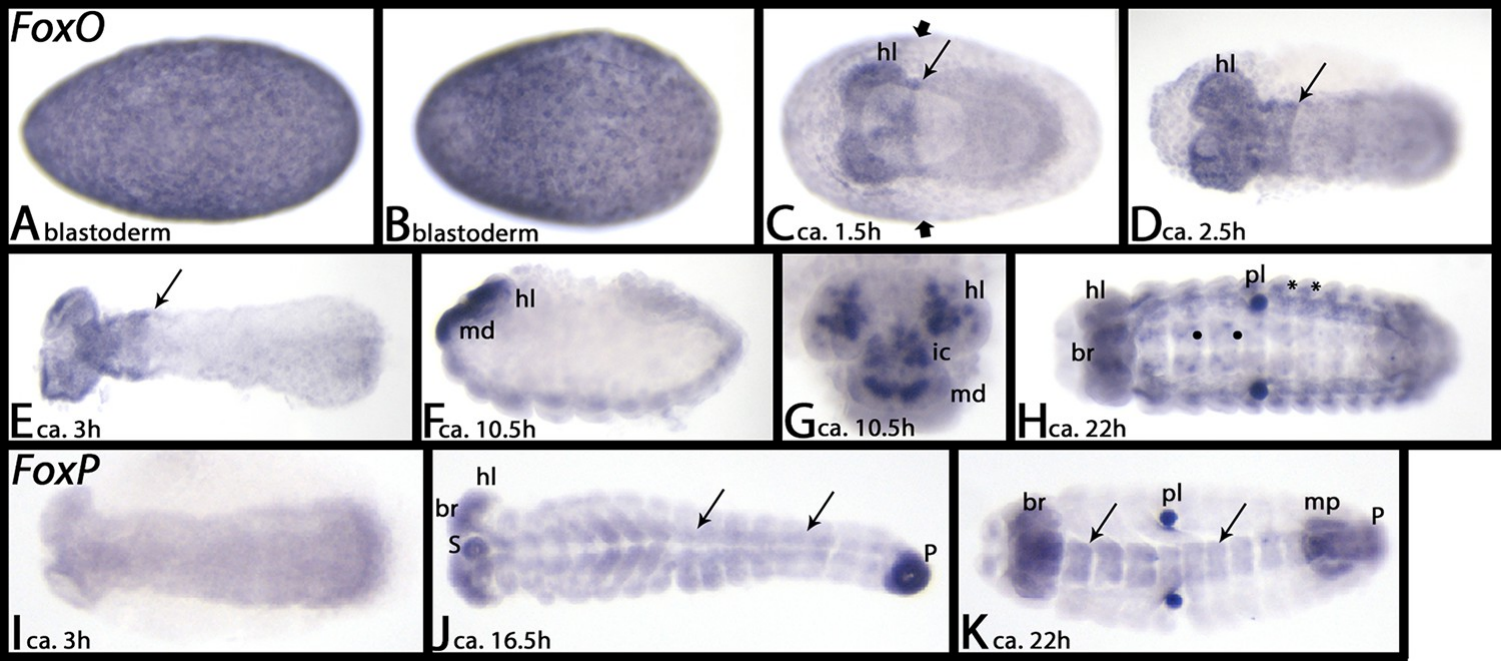
Expression of *Tribolium FoxO* (A-H) and *FoxP* (I-K). In all panels, anterior is to the left, ventral view; except panel F, lateral view. In panel G, anterior is up. Embryos in panels E, H, J and K are flat-mounted. Long arrows in panels C and D mark the posterior border of strong expression in the head. Short arrows in panel C mark posterior border of expression in the extraembryonic tissue. Asterisks in panel H mark expression in the lateral tissue of the trunk segments. Filled circles in panel H mark dot-like expression in the VNS. Arrows in panels J and K point to expression in the VNS. Note the unspecific staining of the pleuropodia in panels H and K. Abbreviations in Table 2.

*Tribolium FoxO* is first expressed ubiquitously (Fig 17A and 17B), but when the germ band forms, expression is restricted to the anterior of the embryo proper, and at lower level in the anterior of the extraembryonic tissue. The expression in the embryo covers all anterior tissue with a sharp posterior border between the mandibular and maxillary segment (Fig 17C–17F, slim arrows). Later, this expression resolves into a complex pattern in the nervous system of the head (Fig 17G), that at later stages is also present in the entire embryo (Fig 17H).

*Glomeris FoxO* is expressed ubiquitously (not shown). However, higher levels of expression are detectable in the labrum, and in the brain (S14C Fig).

**Fig 18:**
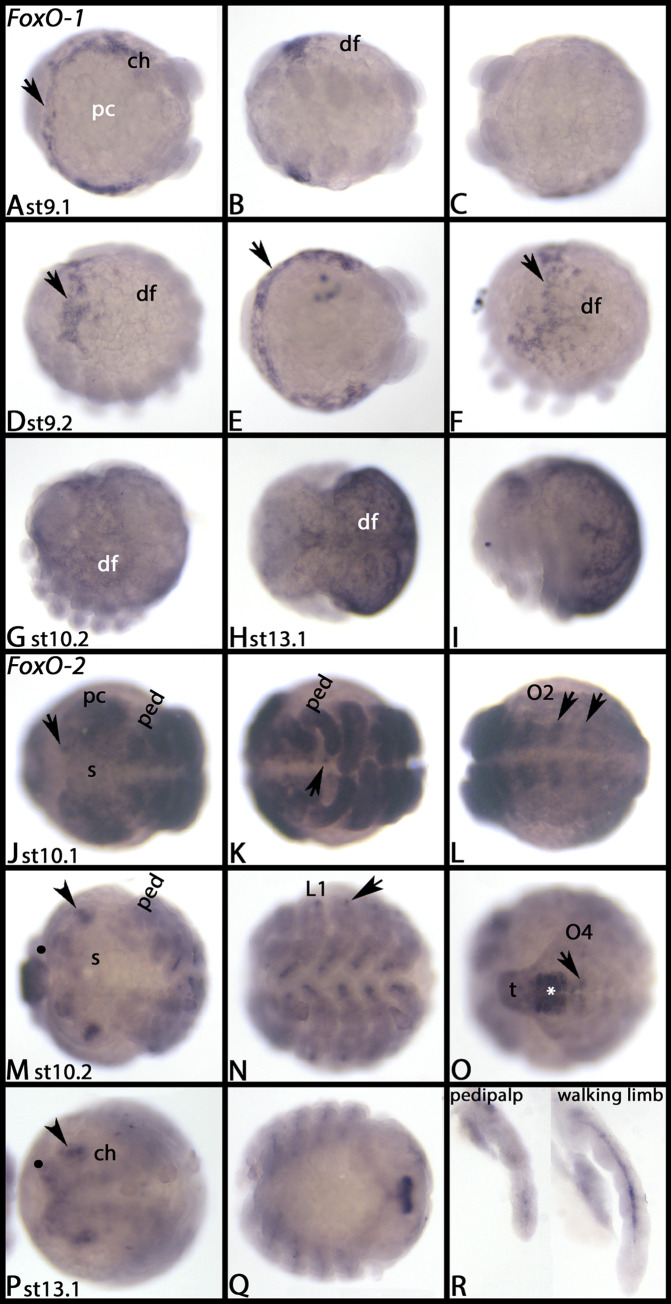
Expression of *Parasteatoda FoxO-1* (A-I) and *FoxO-2* (J-R). In all panels, anterior is to the left, ventral views, except panels D, F, G, I, and R (lateral views), and H (dorsal view). Each row represents the same embryo, except panels G and H. Arrows in panels A, D, E and F point to expression in the interface between the embryo proper and the dorsal field. Arrow in panel J points to dot-like expression in the brain. Arrows in panels K and L point to expression in the VNS. Arrowheads in panels M and P point to lateral expression in the head lobes. Filled circles in panels M and P mark expression anterior in to the mouth. The asterisk in panel O points to strong expression in the VNS of nascent segments; the arrow in panel O points to weaker expression anterior to that. Arrowhead in N points to dot-like expression dorsal to the base of the walking legs. Abbreviations in Table 2. DAPI staining of the embryos shown in this figure are presented in S16 Fig.

*Parasteatoda FoxO-1* is exclusively expressed in the dorsal field and the interface between the embryo proper and the so-called extraembryonic tissue around the head (Fig 18A–18I, arrows). *Parasteatoda FoxO-2* is first expressed ubiquitously and in equal level in all tissue (not shown). From stage 10.1 onwards, stronger expression is visible in ventral segmental patches (Fig 18J–18L, arrows). In stage 10.2 embryos, ubiquitous expression disappears and strong expression is now in the ventral tissue of newly formed posterior segments (Fig 18O, asterisk and arrow), as well as in the mesoderm of walking limbs and pedipalps (Fig 18R), an ectodermal patch of expression at the dorsal base of the appendages (Fig 18N, arrow), two patches of expression in the brain (Fig 18M and 18P, arrowheads) and expression anterior to the mouth (Fig 18M and 18P, filled circles). At stage 13.1, posterior expression is restricted to the proctodaeal region (Fig 18Q). S16 Fig shows DAPI staining of the embryos shown in Fig 18.

**Fig 19:**
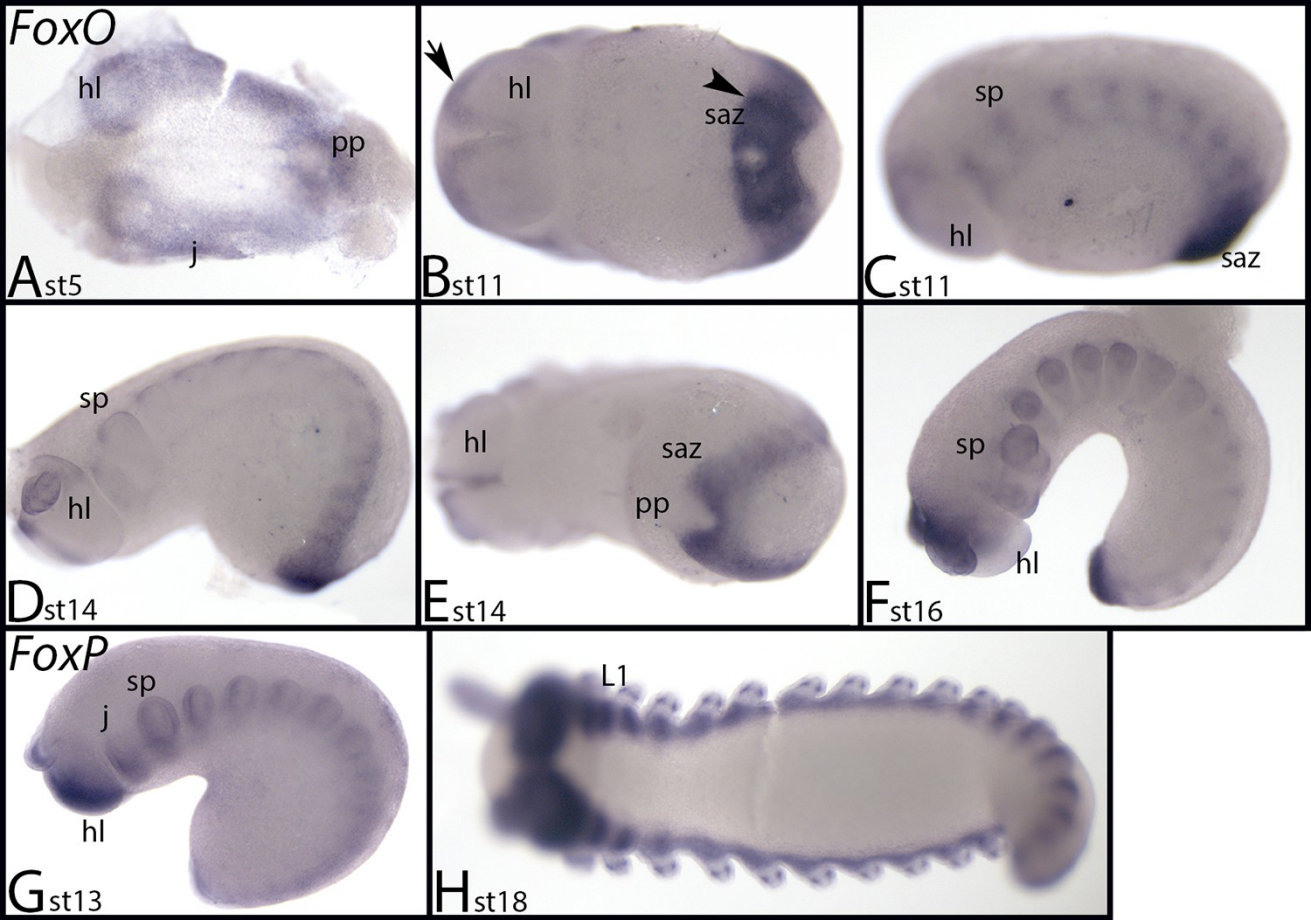
Expression of *Euperipatoides FoxO* (A-F) and *FoxP* (G, H). In all panels, anterior is to the left. Panels A, B, E and H show ventral views; panels C, D, F, and G show lateral views. Arrow in panel B point to expression at the anterior rim of the head lobe. Arrowhead in panel B point to a segmentation gene like ventral stripe of expression in the last formed posterior segment. Abbreviations in Table 2.

*Euperipatoides FoxO* is first expressed ubiquitously, but stronger expression is in the posterior pit and anterior in the head lobes (Fig 19A). Later, expression is in the anterior of the head lobes (Fig 19B, arrow), in the posterior pit, the SAZ (where expression is in a strong transverse stripe, reminiscent of the expression of segmentation genes) (Fig 19B, arrowhead), and in a segmental pattern of weaker transverse stripes in the trunk segments (Fig 19C). In older (more anterior trunk segments) the segmental stripe-pattern disappears and only a dorsal segmental domain remains (Fig 19D and 19E). At stage 16, anterior trunk segments express *FoxO* ubiquitously, while in more posterior segments, the previously described dorsal pattern is still present; the posterior SAZ still expresses *FoxO* at a high level (Fig 19F).

### FoxP

First, *Tribolium FoxP* is not expressed, or is expressed ubiquitously at a low level (Fig 17I). With the beginning of germ band retraction, strong expression appears in the brain, the stomodaeum (including the labrum), the proctodaeum and weakly in the VNS (arrows) (Fig 17J). This pattern remains throughout further development, but expression in the VNS becomes stronger (arrows), and expression in the developing Malpighian tubules appears (Fig 17K).

*Glomeris FoxP* is first expressed in the ocular region (Fig 16I–16L). Later it is also expressed in the form of dots in the mandibles, the maxillae, the antennae (albeit weakly), and the VNS (arrow in panel K) (Figs 16K and S14A Fig). After stage 6, the ventral tissue anterior to the SAZ expresses *FoxP* (Fig 16L, arrow), and faint expression is inside the anal valves (Fig 16L). Expression in the head appendages is restricted to mesodermal tissue; while most of the mesoderm in the mandibles and the maxillae expresses *FoxP*, expression in the antennae, the labrum and the walking limbs is restricted to a ventral and proximal portion of the mesoderm (S14A Fig).

**Fig 20:**
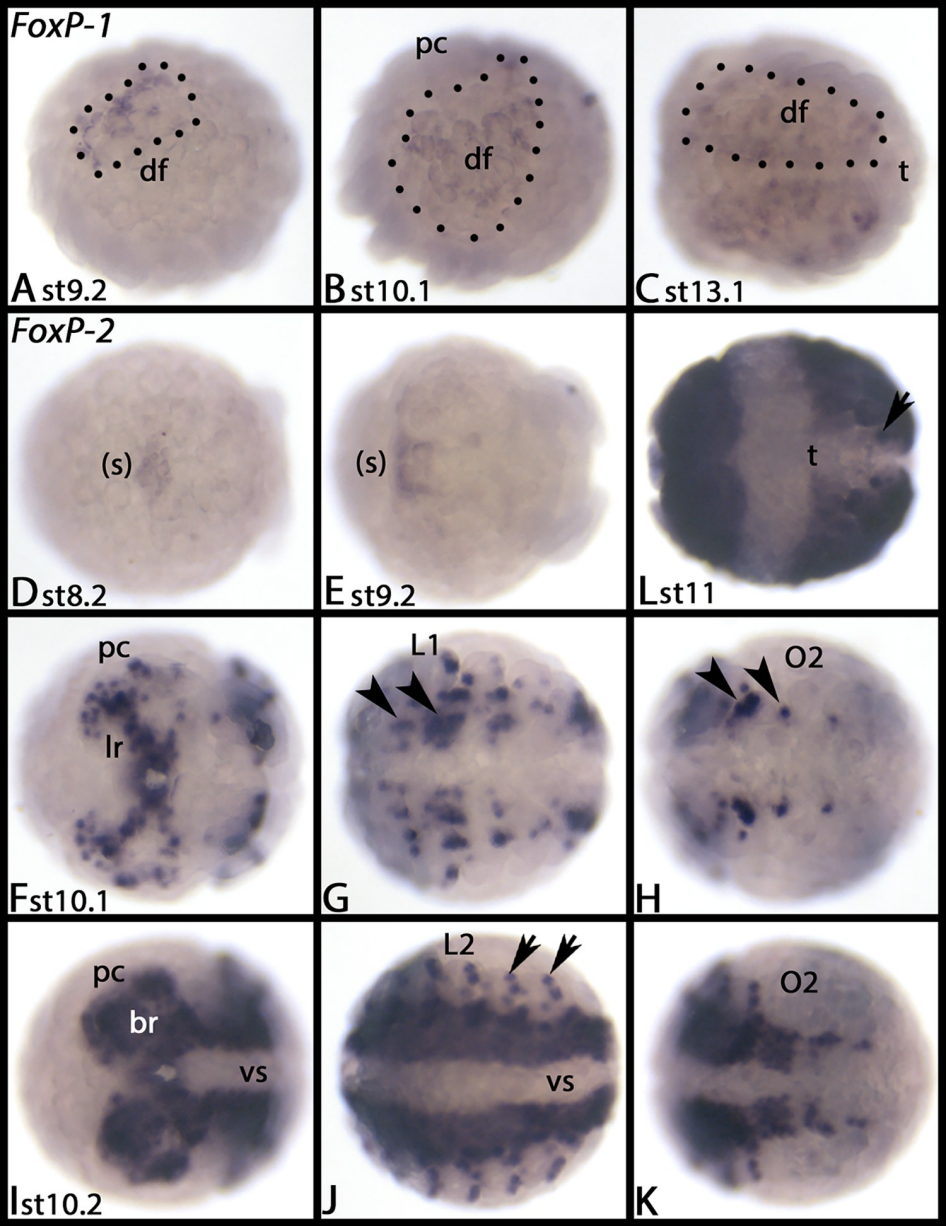
Expression of *Parasteatoda FoxP-1* (A-C) and *FoxP-2* (D-K). In all panels, anterior is to the left. Panels A and B show lateral view; panel C and L show dorsal views. All other panels show ventral views. Dotted lines mark the area of expression in the dorsal field. Arrowheads in panels G and H point to expression in the VNS. Arrows in panel J point to dot-like expression at the dorsal edge of the embryo. Abbreviations in Table 2. DAPI staining of the embryos shown in this figure are presented in S17 Fig.

At stage 9.2, *Parasteatoda FoxP-1* is expressed in the anterior of the dorsal field (Fig 20A). In subsequent stages, this expression extends to the complete dorsal field (Fig 20B), and at stage 13.1, after dorsal closure, expression is in the dorsal of the opisthosoma (Fig 20C).

*Parasteatoda FoxP-2* is first expressed in the primordium of the mouth (Fig 20D and 20E). In subsequent stages, it is expressed in a large number of cells (or cell clusters) in the brain (Fig 20F), the VNS (Fig 20G and 20H, arrowheads) and segmental patches dorsal to the base of the limbs (Fig 20G and 20H, arrows), and later, in most cells of the central nervous system (Fig 20I–20K), except for newly formed posterior segments that express *FoxP-2* later during development (Fig 20L). We did not detect any expression of *FoxP-3*. S17 Fig shows DAPI staining of the embryos shown in Fig 20.

*Euperipatoides FoxP* is expressed ubiquitously but in the limb buds and in the head lobes expression is stronger (Fig 19G). At stage 18, dots of expression appear in the walking limbs and the slime papillae (Fig 19H).

### FoxQ1

*FoxQ1* is not present in the here investigated arthropods, and we were unable to detect expression of *FoxQ1* in *Euperipatoides*.

### FoxQ2

*Tribolium FoxQ2* is exclusively expressed in the head. First, expression in the form of two anterior domains is visible (S18A and S18B Fig). At the end of germ band elongation, these domains resolve into a complex pattern around the stomodaeum (S18C Fig). Expression is now also in the labral buds, the brain and around the mouth (S18C–S18E Fig). The expression and function of *Tribolium FoxQ2* in head and brain development has been reported previously by [60].

*Glomeris FoxQ2* is exclusively expressed in the head where it forms a complex pattern anterior and lateral to the mouth (S3M–S3P Fig). This expression corresponds to the tip of the labrum, the pharynx and a stripe and dot on either side of the labrum. Several aspects of *Glomeris FoxQ2* expression have been reported by [61].

At stages 6/7 to 8.1, *Parasteatoda FoxQ2* is expressed at the anterior margin of the early germ band (S19A and S19B Fig). Later, this domain refines into three patches of expression on either side of the mouth primordium (S19C–S19F Fig, asterisk, open circle, and filled circle). At stage 12, an additional pair of patches appears in the labrum (S19G and S19H Fig). The expression and function in labrum and nervous development have been described previously by [22].

*Euperipatoides FoxQ2* is first exclusively expressed ventrally in the head lobes, anterior to the mouth (S20A and S20B Fig). At later developmental stages, faint expression appears in a small domain ventral of the eyes (S20C and S20D Fig, asterisks) and between the base of the jaws and the slime papillae (S20C and S20D Fig, arrowheads). [61] has also described several aspects of the *Euperipatoides FoxQ2* expression profile.

### FoxT (syn. fd3F)

**Fig 21:**
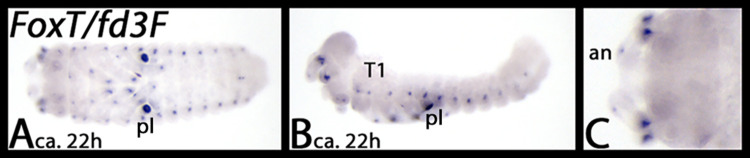
Expression of *Tribolium fd3F*. Panel A ventral view, panel B lateral view and panel C close-up on head, dorsal view. Note the unspecific staining in the pleuropodia in panels A and B. Abbreviations in Table 2.

*Tribolium FoxT* is only expressed in late developmental stages (Fig 21). Dots of expression are in the limbs, and in dorsal tissue along the body, similar as described for *FoxJ1* and *FoxL2*.

We did not find this Fox gene in *Glomeris* and *Parasteatoda*, and we did not detect expression of *FoxT* in *Euperipatoides*.

## Discussion

### Panarthropod Fox genes

The phylogeny and gene content of panarthropod Fox genes have recently been discussed in [28]. According to this analysis, two classes of Fox genes appear to have been lost in Panarthropoda, FoxE and FoxM. Additionally, FoxH has been lost in Arthropoda. A recent analysis, however, claims to have identified a FoxM gene in *Drosophila* (i.e. *CG32006*), a gene that we and others believe is a FoxJ1 ortholog (cf. [62] with [27, 28]). Gene expression analysis of *CG32006*/*FoxJ1* genes supports this interpretation (discussed below). Another potential loss in Arthropoda may concern FoxQ1, but [28] reported a potential *FoxQ1* gene in a scorpion (Fig 1). The onychophoran *Euperipatoides* possesses a large set of Fox genes with single members of all expected classes including *FoxH*. Additionally, *Euperipatoides* possesses an orphan gene that recently has been described as a potential *FoxM* class gene [59]. In the analyses performed by [28], this gene clustered with *FoxM* genes from other animals, albeit with low support. The water bear *Ramazzottius varieornatus*, however, lacks the otherwise conserved genes *FoxD*, *FoxJ1*, *FoxJ2*/3 and *FoxN23*, and also possess no *FoxM* and no *FoxH* (unlike the onychophoran). Overall, the tardigrade thus appears to have retained a much less well conserved set of Fox genes than the onychophoran.

For a pair of *Drosophila* Fox genes that were long considered orphans (*fd3F* and *Crg-1*), [14, 27, 28, 63] identified orthologs in most of the investigated insect species, the water flea *Daphnia*, a scorpion (albeit with weaker support), and the onychophoran *Euperipatoides* (Fig 1). These genes are considered to form a separate group of Fox genes named FoxT [27, 28]. The unique expression of FoxT (*fd3F* and *Crg-1*) genes support the hypothesis that they form a separate group of Fox genes.

### On the function of Fox genes in animals

*FoxA–A conserved factor of metazoan gut development*. The *FoxA* ortholog *forkhead*, the first-identified and founding member of the Fox gene family, is an important player in the development of the ectodermal foregut and hindgut, but also the endodermal posterior midgut and the Malpighian tubules, in the fly *Drosophila* [64–66]. In other arthropods like for example the beetle *Tribolium* [41], the millipede *Glomeris* [42], the spider *Parasteatoda* [43], and the onychophoran *Euperipatoides* [42], *fkh* is also likely involved in gut development (ectodermal fore- and hindgut and endodermal midgut). Expression and thus implied function of *fkh* in the Malpighian tubules appears to be restricted to insects (or possibly Pancrustacea) because in the millipede *Glomeris*, *fkh* is not expressed in the Malpighian tubules [42]. In other ecdysozoans such as the nematode worm *Caenorhabditis* and the priapulid worm *Priapulus*, the function in gut development appears to be conserved as well [67–69]. Outside Protostomia, a general function of FoxA in gut development appears to be conserved in lophotrochozoans [7, 10, 70–77].

*FoxB–A factor of dorsal-ventral body and appendage patterning*. In *Drosophila*, *FoxB* orthologs are expressed in the fully extended germ band stage embryo, in neuroblasts and sensory neurons [15]. A recent study showed that *FoxB* is expressed in conserved patterns in arthropods including *Drosophila* and an onychophoran [20]. *FoxB* is expressed in the ventral sector of the limbs in all panarthropod species, where it is likely involved in dorsal-ventral limb patterning, as functional data from the spider *Parasteatoda* suggest [20]. In addition, *FoxB* is also involved in the transformation of the early germ disc into the bilateral germ band, and thus in dorsal-ventral body patterning [45]. Expression data on *FoxB* in other ecdysozoans is restricted to *Caenorhabditis* (syn. *lin-31*) where it is *inter alia* involved in vulva-development. Interestingly, during this process *lin-31* expression is restricted to a subset of ventral cells, and without the input of *lin-31*, these cells randomly either contribute to the vulva or not, indicating that *lin-31* acts as a binary genetic switch [78, 79]. *FoxB/lin-31* therefore likely acts as a ventral factor. In echinoderms, *FoxB* (syn. *fkh1*) is expressed in the mesenchyme and is involved in gut development. Strongest expression is at the oral (ventral) side of the developing embryo [80–82]. In the hemichordate *Saccoglossus*, *FoxB* is expressed in a complex pattern, but it is striking that *FoxB* is asymmetrically expressed on one side of the blastopore [7]. In vertebrates, *FoxB* (syn. *fkh5*) is expressed in the dorsal ectoderm of the organizer [83, 84]. Given that the dorsal-ventral axis is reversed in chordates vs protostomes (reviewed in [85]), this means that also here *FoxB* is a marker of (ancestrally) “ventral” tissue. These patterns are comparable to the expression along the ventral ectoderm in panarthropod limbs and the ventral cells in *Caenorhabditis*, and therefore, it is possible that *FoxB* is a general discriminator of dorsal versus ventral tissue. If so, this function dates back to the last common ancestor of panarthropods and chordates, the urbilaterian.

*FoxC–A conserved factor of anterior gut and mouth development*. In Drosophila, FoxC (syn. crocodile/croc) is involved in the development of head structures such as the inner head skeleton [86], and this function appears to be conserved in panarthropods, including the species investigated in this study (see also [17, 46, 47, 50, 87]. In the annelid Capitella, FoxC is also dominantly expressed in mesodermal structures in the head and around the foregut [9]. Similarly, in the leech Helobdella austinensis, FoxC is expressed in the musculature associated with the developing proboscis [73]. In a brachiopod larva, FoxC is expressed in the anterior of the archenteron and associated structures [88]. In the hemichordate, FoxC is involved in the development of anterior structures such as the proboscis and the anterior mesoderm [7]. In vertebrates, FoxC genes are expressed in the mesoderm and are involved in head development as well, including the development of pharyngeal structures [89] (and references therein). In the cnidarian Nematostella that like all cnidarians lacks mesoderm, FoxC is expressed in the pharyngeal endoderm and the first-developed mesenteries, but not the body endoderm or the other mesenteries [90]. Given that the mesoderm may have evolved from the endoderm in the diploblast ancestor [91], this expression may be homologous to that of anterior mesodermal structures in bilaterian animals.

Altogether, these expression patterns imply a specific function of FoxC genes in head mesoderm development that likely dates back to the last common ancestor of all eumetazoan animals.

*FoxD–A conserved factor of ecdysozoan nervous system patterning*. *Drosophila FoxD* is expressed in a subset of procephalic neuroblasts, some neuroblasts in the VNS, and sensory organs in the trunk and in the brain [15, 92]. This pattern is conserved in *Tribolium*, *Glomeris*, and *Parasteatoda*, where *FoxD* is strongly expressed in the brain and the VNS. In the onychophoran, however, there is only expression in the brain, but not along the VNS, suggesting that this latter aspect of *FoxD* is restricted to Arthropoda.

In *Caenorhabditis*, *FoxD* (syn. *unc-130*) [16] is involved in axon guidance and the specification of neuronal tissues [93, 94], as well as in dorsal-ventral patterning of the postembryonic mesoderm [95, 96]. In other protostomians such as brachiopods and annelids, *FoxD* is expressed in both mesodermal and ectodermal derivatives [88, 97]. In deuterostomes like echinoderms, *FoxD* appears to be predominantly expressed in ectodermal tissue [8, 98]. In ascidians, *FoxD* is involved in the patterning of mesodermal and endodermal tissue, in notochord induction, and in the patterning of the animal-vegetal body axis [99–101]. In the hemichordate *Saccoglossus*, and the cephalochordate *Branchiostoma*, *FoxD* is expressed in mesodermal tissues [7, 102]. In *Nematostella*, *FoxD* is first expressed at the aboral pole suggesting a role in patterning the oral-aboral axis. Later it is expressed at the base of the tentacles [90].

In summary, this suggest that *FoxD* played a role in both ectoderm and mesoderm patterning in the last common ancestor of at least Bilateria, and that lineage-specific losses of mesodermal or ectodermal expression/function happened frequently in different evolutionary lineages (see also [88]). In panarthropods, and possibly in ecdysozoans as a whole, *FoxD* appears to be involved in the development of the nervous system.

*FoxF–A conserved factor of visceral mesoderm development*. In *Drosophila*, *FoxF* is involved in the formation of the visceral mesoderm and the midgut [103–106]. Expression in *Tribolium*, *Glomeris*, *Parasteatoda*, and *Euperipatoides* appears to be mainly in mesodermal tissue of the trunk suggesting that the function of *FoxF* in the patterning of the visceral mesoderm, or at least part of it, is conserved in Panarthropoda. Data from other ecdysozoans are restricted to *Caenorhabditis*. Here, the single FoxC/FoxF gene (syn. *Let-381*) is involved in the development of non-muscle mesodermal tissue [107, 108]. In other protostomes like brachiopods, planarians and molluscs the function in visceral muscle development appears to be conserved [9, 88, 109], and so is this function in deuterostomes such as echinoderms, ascidians, hemichordates and vertebrates (e.g. [7, 110–112]).

Summarized, our current knowledge on FoxF genes strongly suggests a conserved function in visceral mesoderm development in bilaterian animals. Interestingly, there is no clear ortholog of *FoxF* in cnidarians which could be correlated to the lack of clear-cut mesoderm in these animals (e.g. [113, 114]).

*FoxG–A conserved factor in arthropod segmentation*, *and bilaterian brain and ciliary nervous system development*. The *Drosophila FoxG orthologs sloppy-paired 1* (*slp1*) and *sloppy-paired 2* (*slp2*) play redundant but essential functions in the segmentation gene cascade where they act as segment-polarity and pair-rule genes [115–117]. The segmentation gene function of *FoxG* has been investigated in various other arthropods and an onychophoran, suggesting that it is conserved in arthropods but not onychophorans (e.g. [55, 39, 55, 118–120]). In *Drosophila*, *slp1*, but not *slp2*, also acts as an important factor of early head development [121]. Interestingly, our recent phylogenetic analysis revealed that the previously identified *fd19B* gene [14] represents a third FoxG-class gene in *Drosophila* [28, 62] (Fig 1 and S1 Fig). Its expression pattern suggests that it may contribute to the function of *slp1* in head development.

In *Caenorhabditis*, *FoxG* (syn. *fkh-2*), is involved in the development of a subset of ciliary neurons [122]. In annelid larvae, *FoxG* is expressed in the brain and a subset of cells of the ciliated bands that are in close proximity to the locomotory cilia [123, 124]. In a planarian, it is expressed in the brain [125]. In echinoderms, *FoxG* is expressed in the ciliary bands where it is likely involved in patterning the underlying nervous system [8, 126]. In hemichordates, *FoxG* is expressed in the anterior of the developing embryo that harbors the brain and, at earlier developmental stages, it is expressed in close proximity to the ciliated band [7]. In cephalochordates, *FoxG* is involved in brain development including the development of nerves that are associated with ciliated sensory receptors [127]. In vertebrates, *FoxG* is involved in the development of the telencephalon (e.g. [128]).

The available data suggest that the role of *FoxG* genes in segmentation and head development is restricted to arthropods, and that the ancestral function of *FoxG* was likely in brain development including the development of the nervous system associated with ciliary cells.

*FoxH*. In the onychophoran, *FoxH* is expressed transiently in the head lobes where the brain will form. Data on FoxH from other animals is extremely scarce. In chordates, it appears to be involved in the development of left-right asymmetries of the main body axis [129].

*FoxJ1 –A conserved factor of motile cilia development*. Primary cilia (i.e non-motile cilia) and motile cilia are known from a wide range of animals. In ecdysozoans, however, only the sperm and the chordotonal organs (bipolar neurons) possess motile cilia (e.g. [130]). *FoxJ1* appears to be a master gene of motile cilia development as it is not only needed but also sufficient to induce motile cilia development [131, 132]. Both primary cilia and motile cilia are under control of another transcription factor, the *Regulatory factor X* (*Rfx*) (e.g. [133]). In *Drosophila* and other ecdysozoans, the function of Rfx in primary cilia development appears to be conserved (e.g. [134, 135]). The suggested loss of *FoxJ1* in ecdysozoans as a regulator of motile cilia was therefore not very surprising [24].

Recent studies, however, showed that the previously uncharacterized *Drosophila* forkhead-box gene *CG32006* likely represents a FoxJ1-class gene, and that *FoxJ1* genes are present in at least most of Panarthropoda [27, 28]. We found gene expression data of *CG32006/FoxJ1* in the Berkeley Gene Expression Patterns database (BDGP, [136, 137]). *Drosophila FoxJ1* is expressed in the form of two distinct domains in the anterior head and numerous small dots of expression along the body, a pattern that could correlate with the development of bipolar neurons. In this context, it is interesting to note that Rfx-dependent genes that are supposed to be expressed in all ciliated cells (e.g. *CG31036* [134]) are indeed expressed in a larger number of cells (or cell clusters) than *FoxJ1* (*CG32006*) (see BDGP), which would be in line with a potentially exclusive function of *FxoJ1* in the development of motile cilia.

The here reported expression profiles of *FoxJ1* in *Tribolium* and *Parasteatoda* are comparable with those of *Drosophila FoxJ1*. Detection of *FoxJ1* transcripts in the “embryonic” transcriptome of *Euperipatoides* suggests late expression in developmental stages that have been included in mRNA extraction for transcriptome sequencing, but that are too late in development to work in whole mount in-situ hybridization experiments. Note in this context that expression of another potentially conserved motile cilia-specific Fox gene, *FoxT*, was not detected in the investigated embryonic stages of the onychophoran either (discussed below).

Data on FoxJ expression outside Ecdysozoa support the idea that *FoxJ1* is a conserved factor of motile cilia development: In annelids, *FoxJ1* is expressed in association with ciliary and sensory cells [138], and in a sea urchin, it is expressed in the area of the apical plate and the ciliated bands [8]. In hemichordates, *FoxJ1* is also involved in the development of the ciliated band and the apical organ [7]. In vertebrates, *FoxJ1* is associated with the development of ciliated cells (e.g. [131, 139, 140]). In cnidarians such as *Nematostella*, *FoxJ1* is expressed in the ciliated apical organ [141], and it has been shown that *FoxJ1* is also present in the earliest animals, sponges and choanoflagellates [142, 143].

In summary, as previously suggested by [132], *FoxJ1* appears to be a conserved regulator of motile cilia cell development, and as we show here, this may even be the case in arthropods.

*FoxJ2 (syn*. *FoxJ2/3)*. The expression patterns of *Euperipatoides*, *Glomeris*, and *Parasteatoda* are diverse and thus do not allow speculation on conserved functions. In the latter two species, *FoxJ2* is expressed ubiquitously, and thus not very informative. Other data on *FoxJ2* expression are scarce. In *Saccoglossus* expression of *FoxJ2* could not be detected [7]. In the frog *Xenopus*, *FoxJ2* is first expressed ubiquitously, but at later developmental stages it is expressed in the notochord and the ventral region of the neural tube [144]. In mouse, *FoxJ2* regulates meiosis in spermatogenesis [145]. *FoxJ2* is also involved in some forms of cancer (e.g. [146]). In vertebrates, *FoxJ3* appears to be a neurogenic factor [147], and this is also the case in the cnidarian *Hydra*, where *FoxJ3* appears to be involved in neurogenesis [148].

*FoxK–A potentially conserved factor of cell cycle control*. *FoxK* is present and expressed ubiquitously in all investigated panarthropod species, including *Drosophila* [136, 137]. Functional studies have shown that at later developmental stages, *FoxK* is involved in the formation of the midgut in *Drosophila* [149]. In *Saccoglossus*, expression of *FoxK* is ubiquitous in the ectoderm, although expression is weaker in the ciliary band [7]. It has recently become clear that FoxK class genes are involved in cell cycle control and cancer (reviewed in [150]). Although information on *FoxK* expression and function is scarce, it appears likely that it is involved in cell metabolism and/or cell cycle control (cf. ubiquitous expression of FoxN class genes and their function in cell cycle control).

*FoxL1 –A potentially conserved factor of gut (and associated structures) development*. *Drosophila FoxL1* is expressed in a posterior and ventral region of the blastoderm that then invaginates to form part of the posterior mesoderm. Later, pairs of segmental clusters of *FoxL1*-expressing cells appear in the trunk [15, 151]. It has been shown that *FoxL1* plays a role in organ placement, and that in knock-out fly embryos, various organs like the germ cells and the Malpighian tubules fail to position correctly [151]. In *Tribolium*, the expression of *FoxL1* is conserved in the developing hind and midgut and in segmental dots in the trunk. In *Glomeris*, *Parasteatoda* and *Euperipatoides*, we find a comparable early posterior domain of expression that demarcates the hindgut. The segmental expression, however, is not present in *Glomeris*, and the anterior expression in the brain and the mouth/pharynx seen in these species is not present in *Drosophila* and *Tribolium*. In *Saccoglossus* and the shark *Scyliorhinus*, *FoxL1* is expressed in the developing gill slits [7, 89]. In vertebrates, *FoxL1* is an important component of gut development [152, 153].

It appears that FoxL1 is a conserved factor in gut development, including the development of associated structures such as the pharynx, Malpighian tubules, and gill slits.

### FoxL2

*FoxL2* is absent in *Drosophila* but is present in most other panarthropods (Fig 1). However, in the spider and the onychophoran, we could not detect expression. In *Tribolium* and *Glomeris*, *FoxL2* is expressed late during development and is mainly restricted to dorsal segmental tissue of the trunk segments. In the planthopper *Nilaparvata*, expression of *FoxL2* is female-specific and has a function in chorion development [27, 154]. Besides the potentially conserved function of *FoxL2* in egg-development, we assume that there is a conserved function of this gene in patterning dorsal tissue (possibly muscles) in at least mandibulate arthropods.

In the leech *Helobdella*, *FoxL2* is expressed in developing muscle tissue as well [73]. In the echinoderm *Strongylocentrotus*, *FoxL2* is not detectable or is expressed ubiquitously at low levels early during embryogenesis [8] (their Supporting information). In *Saccoglossus*, *FoxL2* is present, but transcripts could not be detected [7]. In vertebrates, *FoxL2* is a known factor of female gonadogenesis (reviewed in [155]), a function that could be conserved in the oyster *Crassostrea* [156]. Interestingly, in both groups of animals there is an anti-sense transcript of *FoxL2* that is likely involved in the regulation of *FoxL2* sense transcripts [157, 158]. However, we did not detect expression using sense-probes neither for *Glomeris* nor for *Tribolium FoxL2* (data not shown). In the sponge *Suberites* expression is ubiquitous [13].

*FoxM*, *FoxN14*, *and FoxN23 –A trio of cell cycle controlling genes*. *FoxM* appears to be lost in arthropods (Fig 1). In the onychophoran, *FoxM*, *FoxN14* and *FoxN23* all are expressed in a complex dynamic pattern, suggesting a function in cell cycle control [59]. In other arthropods, expression of FoxN genes is ubiquitous [59], a pattern that is in line with a function in mitotic cells and thus cell cycle control. However, the available panarthropod data suggest that the situation in *Drosophila*, where FoxN *genes* are differentially expressed in various tissues, is derived [159–162].

The role of *FoxM*, and FoxN genes in cell cycle control is also conserved in vertebrates (reviewed in [163, 164]) [165–167], suggesting that a function of these genes in controlling the cell cycle is conserved among at least Bilateria.

*FoxO*. Expression profiles of panarthropod *FoxO* orthologs are diverse. In *Drosophila*, *FoxO* is expressed maternally, but soon after fertilization, transcripts disappear until at stage 11 when *de novo* expression appears in ectodermal and endodermal tissue. Expression levels in ventral tissue of the trunk and the head are low except for the labrum that strongly expresses *FoxO* [14]. In *Tribolium*, zygotic expression is mainly restricted to the head region and later in the developing brain and nervous tissue in the head. In *Glomeris*, expression is ubiquitous. Of the two *Parasteatoda* FoxO orthologs, *FoxO1* is expressed in the dorsal field, and *FoxO2* is expressed in complex patterns during development. Finally, onychophoran *FoxO* is expressed in a complex pattern as well; some aspects of its expression may be conserved between the spider and the velvet worm.

In *Caenorhabditis*, *FoxO* (syn. *daf16*) is a mediator of *dauer formation* (halting development) and aging [168, 169], mediates insulin-like metabolic signaling and stress resistance, and is involved in learning, memory, and regeneration [170], many of the functions that are conserved in *Drosophila*, other insects such as the silkworm *Bombyx* [171–173] and mouse (reviewed in e.g. [174]). In *Saccoglossus*, *FoxO* was not detectable in early development [7]. In *Hydra*, *FoxO* is involved in the regulation of stem cell proliferation and antimicrobial peptides that are components of the immune system and the microbiome [175, 176]. The only expression data from lophotrochozoan species come from the leech *Helobdella austinensis* where its two FoxO genes both are expressed in complex patterns [73], and the planarian *Schmidtea mediterranea* where the gene appears to be expressed ubiquitously [62].

Altogether, FoxO genes appear to represent important and conserved factors in regulating animal metabolism. This is in line with the often-ubiquitous patterns of *FoxO* during development.

*FoxP—A conserved factor of bilaterian nervous system development*. In *Drosophila*, *FoxP* is expressed in the yolk cytoplasm as well as in the central nervous system where it starts with the occurrence of segmental groups of *FoxP*-expressing cells along the ventral midline. Later, the complete central nervous system expresses *FoxP* [14]. Functional studies have shown that FoxP indeed is needed for developmental processes in the nervous system (e.g. [177–179]). In the honey bee *Apis mellifera*, and other bees, *FoxP* is also expressed in the brain [180, 181]. This pattern is conserved in the here investigated arthropods and in the onychophoran. In all species, at least one paralog is expressed in the brain and the VNS. In the spider, one of the two paralogs, *FoxP1* is expressed in the dorsal field. Most probably this pattern represents a neo-functionalization after the duplication, whereas the second paralog, *FoxP2* fulfils the ancestral function in nervous system patterning. Although the pattern is less clear in the onychophoran, *FoxP* is strongly expressed in the brain and in the region of the VNS. The function of *FoxP* in nervous system patterning is thus likely conserved in Panarthropoda.

In *Saccoglossus*, *FoxP* is predominantly expressed in ectodermal tissue, suggesting that also here, *FoxP* may be involved in nervous system patterning [7], and in vertebrates, *FoxP* is known to be a key player of nervous system development (e.g. [182–184]), suggesting that *FoxP* is a universal factor of bilaterian nervous system development. *FoxP* genes have been identified in cnidarians and even sponges [142, 185], but expression or functional data are not available leaving the question open of whether the suggested function of FoxP as a neuronal gene may date back even beyond Bilateria.

*FoxQ1 –A conserved factor of pharynx development*. Although we identified a *FoxQ1* gene in the onychophoran *Euperipatoides* (Fig 1) [28], we could not detect expression in the developmental stages that we investigated. In urochordates, hemichordates, cephalochordates, and vertebrates, *FoxQ1* is specifically expressed and functions in the development of pharyngeal structures (e.g. [7, 24, 144, 186]). Interestingly, in an annelid, *FoxQ1* is expressed in the pharynx as well [9], suggesting that the ancestral function of *FoxQ1* in development is restricted to the development of the pharynx.

*FoxQ2 –A highly conserved factor of anterior development*. *FoxQ2* is a factor of the so called anterior gene regulatory network (aGRN) that appears to be highly-conserved in all Bilateria, and even diploblasts (e.g. [123, 138, 141, 187]). Consequently, in all hitherto investigated arthropods, *FoxQ2* is expressed in the anterior of the developing embryo including the anlagen of the pharynx and the anterior procephalic neuroectoderm [14, 22, 60, 61, 188, 189]. Given the conserved expression patterns of other anterior patterning genes such as *six3* and *orthodenticle* (*otd*) the anterior patterning GRN as a whole, or at least key-components of it, appear to be conserved in panarthropods (e.g. [47, 61, 188, 190–192]), and indeed all groups of animals (e.g. [190, 193–195]), although, surprisingly, *FoxQ2* has been lost in placental mammals (e.g. [5, 24]).

*FoxT–A potentially conserved factor of hexapod chordotonal sensory cell development*. A new class of Fox genes, FoxT, was recently identified [27, 28]. In *Drosophila*, two genes belong to this class, *fd3F* and *Crg-1* (Fig 1 and S1 Fig). *fd3F* is first expressed ubiquitously, but from stage 12 onwards expression is exclusively in cell clusters along the ventral and lateral side of the embryo [14, 63, 196]. These cell clusters correspond to chordotonal (Ch) sensory organs and their precursors, and it has been shown that *fd3F* regulates specification of this group of ciliated neurons, while the other group of ciliated neurons, the external sensory (ES) neurons, do not express *fd3F* [63, 196]. The function of *fd3F* is thus similar to that of another Fox gene, *FoxJ*, and it has been suggested that *fd3F* may represent a highly-derived FoxJ-class gene [63]; recent phylogenetic analyses, however, do not support this idea (discussed above) [27, 28].

Our data suggest that the function of *fd3F* is conserved in at least insects, because the expression pattern of *Tribolium FoxT/fd3F* are very similar to that in *Drosophila*. We could not detect specific expression of *Euperipatoides FoxT/fd3F*, which could be explained by the relatively late development of Ch neurons (cf. expression in insects), and gene expression studies in late stages of onychophorans are problematic.

*Drosophila Crg-1* is expressed in the adult head, and is involved in steering the circadian rhythm of the fly [197]. [28] suggested that *fd3F* and *Crg-1* are the result of a duplication event of FoxT in *Drosophila*.

The single FoxT-type gene of the planthopper *Nilaparvata* appears to be exclusively expressed in the testis of late male nymphs and adult males [27]. This finding could explain why we were not able to detect expression of *FoxT* in embryos of the onychophoran *Euperipatoides*. The lack of *FoxT* earlier in the development of *Nilaparvata*, however, suggests that the pattern (and thus function) of *FoxT* reported in *Drosophila* and *Tribolium* may be restricted to holometabolous insects.

## Supporting information

S1 Table Primer sequences. (XLSX)

S2 Table Accession numbers. (XLSX)

S1 Fig The complement of panarthropod Fox genes investigated in this paper and of the model arthropod *Drosophila melanogaster*. Each box indicates one paralog of a given Fox-class gene. Horizontal black bars indicate gene loss.
(TIF)

S2 Fig Expression of *Tribolium FoxA*. In all panels, anterior is to the left. A Ventral view. B Lateral view. C Dorsal view. D Dorsal view of posterior end of embryo. The short arrows in A-C indicate expression laterally in the head lobes. The long arrow in A marks expression along the ventral midline. Asterisks mark unspecific staining of the pleuropodia. Abbreviations in Table 2.
(TIF)

S3 Fig Expression of *FoxA* (A-D), *FoxC* (E-H), *FoxG* (I-L), and *FoxQ2* (M-P). In all panels, anterior is to the left, ventral views (except panels D and L, ventral lateral). The arrow in panel D point to expression in the VNS. The asterisk in panel I marks the mandibular segment. Arrows in panels K and L mark lateral dots of expression. Abbreviations in Table 2.
(TIF)

S4 Fig Early expression of *FoxA-1* (A-F) and comparison of expression of *FoxA-1* (F) and *FoxA-2* (G) in the dorsal field. Note that *FoxA-1*, but not *FoxA-2* is expressed in the dorsal field. The x in panel A marks the center of the germ disc that expresses *FoxA1*. Abbreviations in Table 2.
(TIF)

S5 Fig Expression of *Parasteatoda FoxA-1*. In all panels, anterior is to the left, ventral view. Panels A, D and G, view of anterior with head. Panels B, E and H view of middle part with walking limbs. Panels C, F, and I view of opisthosoma. Asterisks in panel G mark expression in the chelicerae. A´-I´ represent DAPI staining of the embryos shown in A-I. Each row (e.g. A-C) represents the same embryo. Abbreviations in Table 2.
(TIF)

S6 Fig DAPI staining of the embryos shown in Fig 2. Abbreviations in Table 2.
(TIF)

S7 Fig Expression of *Euperipatoides FoxA*. In all panels, anterior is to the left, ventral views, except panel B, lateral view, dorsal up. A´-C´ represent DAPI staining of the embryos shown in A-C. Arrows in panels B and C mark expression along the ventral margin of the embryo proper. Abbreviations in Table 2.
(TIF)

S8 Fig Expression of *FoxB*. Expression of *Tribolium FoxB1* and *FoxB2* (A-F), *Glomeris FoxB* (G-J), *Parasteatoda FoxB* (K-M), and *Euperipatoides FoxB* (N-P). In all panels, anterior is to the left (except panel M, ventral to the left). All panels represent ventral views (except panels M, N and P, lateral views). Narrow arrows in panels C, E, F, L, N and P point to the ventral nervous system. The asterisks in panels C and F mark unspecific signal in the pleuropodia. The arrow in panel J points to the midline. The arrow in panel K points to expression around the mouth (stomodaeum). The asterisk in panel K marks expression in the posterior end of the embryo. The arrow in panel O points to expression in the ventral tissue of the appendage. Abbreviations in Table 2.
(TIF)

S9 Fig Expression of *Tribolium FoxC*. In all panels, anterior is to the left, ventral view. Embryos in D-F are flat-mounted. Long arrows in panels C-F mark expression in the VNS. Short arrow in F points to expression in dorsal tissue that could contribute to the heart. Asterisks in F mark unspecific staining in the pleuropodia. Abbreviations in Table 2.
(TIF)

S10 Fig DAPI staining of the embryos shown in Fig 6. Abbreviations in Table 2.
(TIF)

S11 Fig Additional aspects of Glomeris *FoxF* expression. Anterior is to the left, ventral views. Abbreviations in Table 2.
(TIF)

S12 Fig DAPI staining of the embryos shown in Fig 8. (TIF)

S13 Fig DAPI staining of the embryos shown in Fig 9. Abbreviations in Table 2.
(TIF)

S14 Fig Expression of *Glomeris FoxP* (A), *FoxJ1* (B) and *FoxO* (C), additional aspects. Anterior views. Arrowhead in panel B points to dot of expression in the lateral head. Arrowhead in panel C points to expression in the labrum. Abbreviations in Table 2.
(TIF)

S15 Fig DAPI staining of the embryos shown in Fig 13. Abbreviations in Table 2.
(TIF)

S16 Fig DAPI staining of the embryos shown in Fig 18. Abbreviations in Table 2.
(TIF)

S17 Fig DAPI staining of the embryos shown in Fig 20. Abbreviations in Table 2.
(TIF)

S18 Fig Expression of *Tribolium FoxQ2*. In all panels, anterior is to the left, ventral view. Embryos are flat-mounted, except embryo shown in panel A and E. The out-of-focus signal in the center of the embryo shown in panel E is in the pleuropodia that stain unspecific. Abbreviations in Table 2.
(TIF)

S19 Fig Expression of *Parasteatoda FoxQ2*. In all panels, anterior is to the left, anterior view, except panels B, lateral view. A´-H´ represent DAPI staining of the embryos shown in A-H. In all panels, asterisks, filled circles and open circles mark corresponding domains of expression during development. Abbreviations in Table 2.
(TIF)

S20 Fig Expression of *Euperipatoides FoxQ2*. In all panels, anterior is to the left. Panels A and B, ventral view; panel C, lateral view, dorsal up; panel D, dorsal view. A´-D´ represent DAPI staining of the embryos shown in A-D. Asterisks in panels C and D mark faint expression ventral to the eyes. Arrowheads in panels C and D point to expression in the interface between jaws and slime papillae. Abbreviations in Table 2.
(TIF)
