# Supplementary figures and images for: A comprehensive study of arthropod and onychophoran Fox gene expression patterns
Source: PLoS One. 2022 Jul 8;17(7):e0270790. doi: 10.1371/journal.pone.0270790 (PMC9269926; doi:10.1371/journal.pone.0270790)

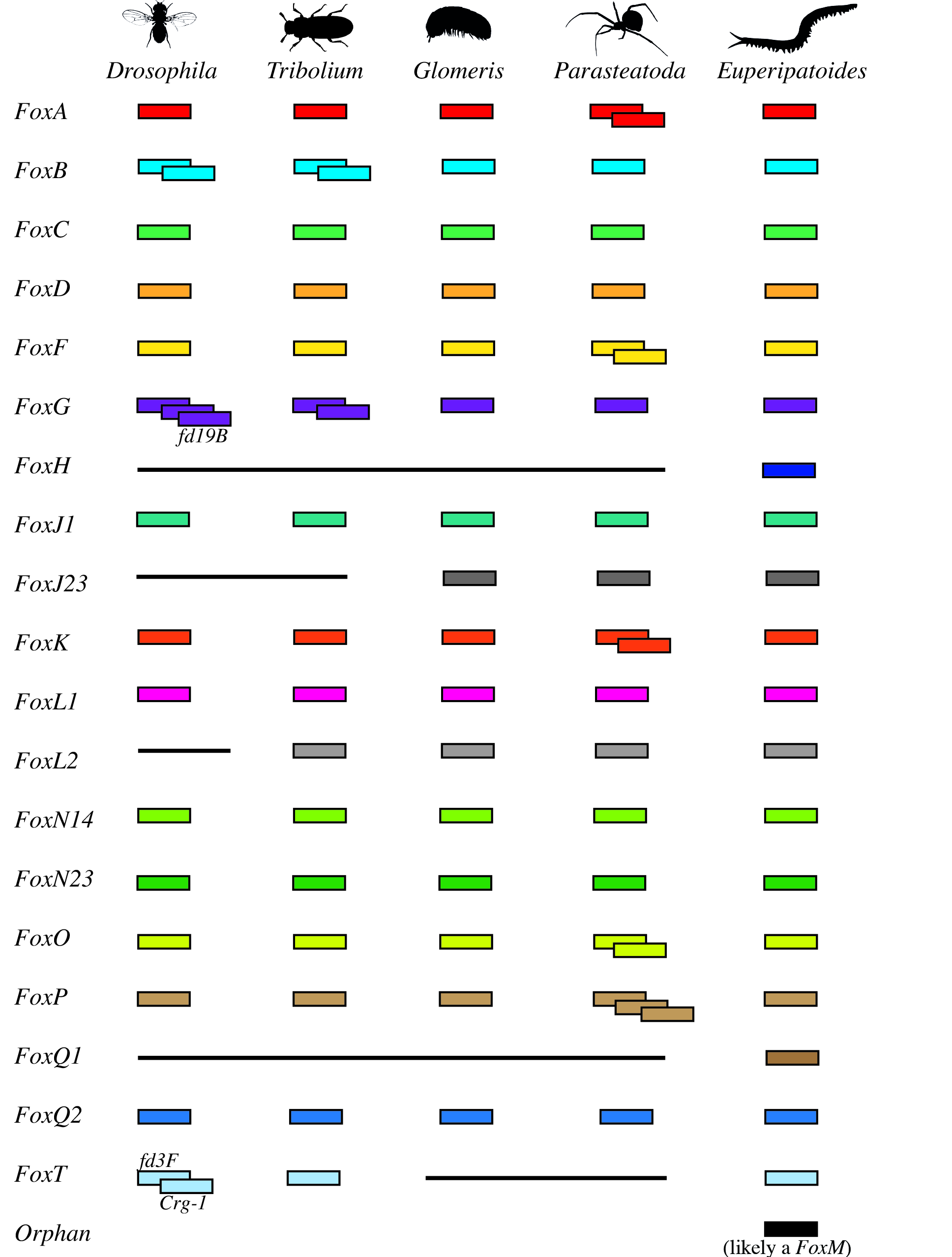

Supplement: S1 Fig — Each box indicates one paralog of a given Fox-class gene. Horizontal black bars indicate gene loss. (TIF) [file pone.0270790.s003.tif]

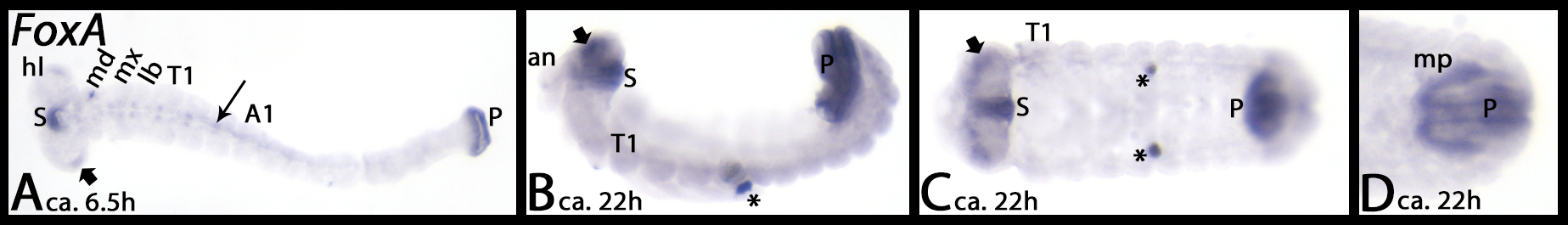

Supplement: S2 Fig — In all panels, anterior is to the left. A Ventral view. B Lateral view. C Dorsal view. D Dorsal view of posterior end of embryo. The short arrows in A-C indicate expression laterally in the head lobes. The long arrow in A marks expression along the ventral midline. Asterisks mark unspecific staining of the pleuropodia. Abbreviations in Table 2. (TIF) [file pone.0270790.s004.tif]

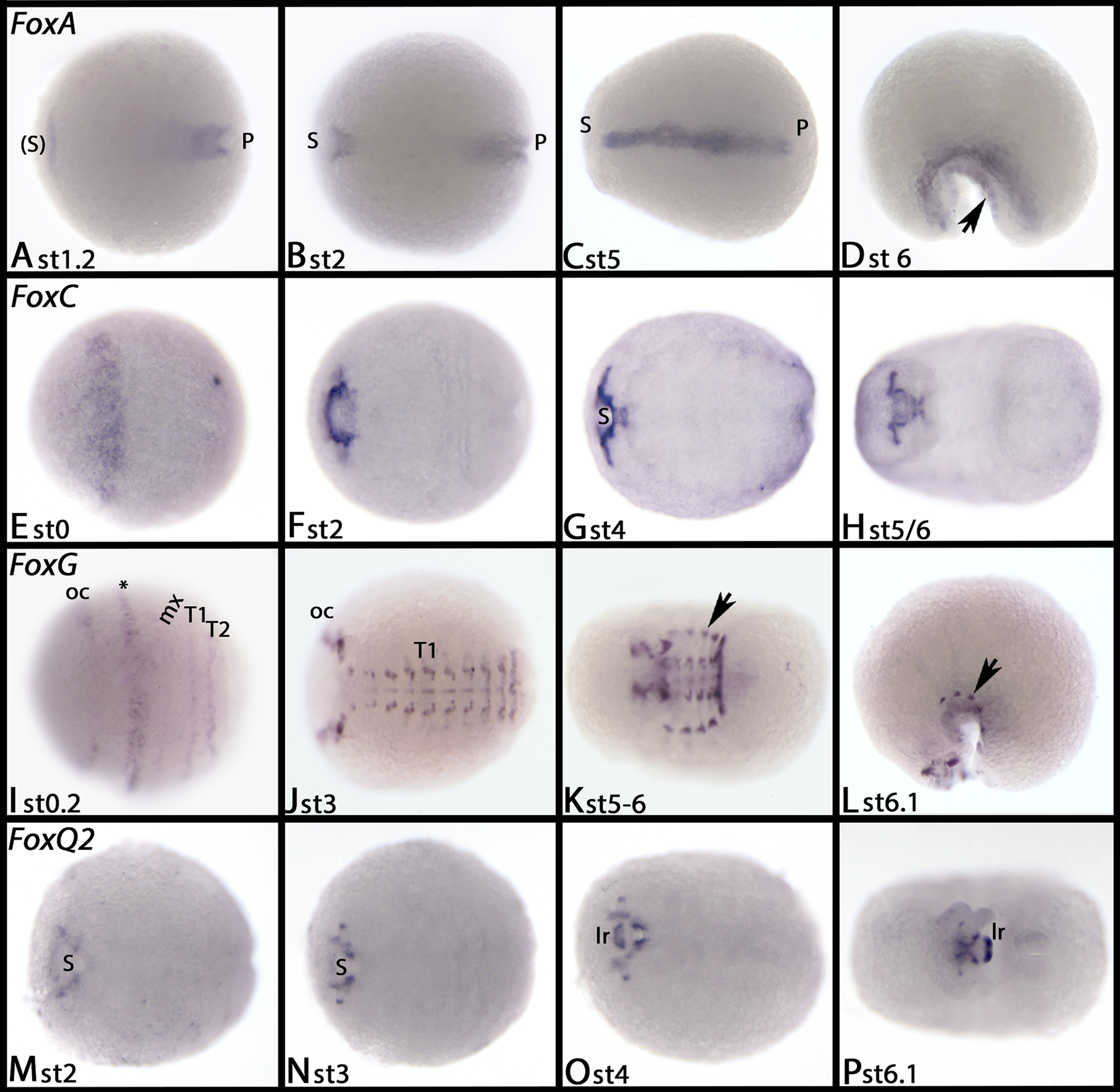

Supplement: S3 Fig — Expression of FoxA (A-D), FoxC (E-H), FoxG (I-L), and FoxQ2 (M-P). In all panels, anterior is to the left, ventral views (except panels D and L, ventral lateral). The arrow in panel D point to expression in the VNS. The asterisk in panel I marks the mandibular segment. Arrows in panels K and L mark lateral dots of expression. Abbreviations in Table 2. (TIF) [file pone.0270790.s005.tif]

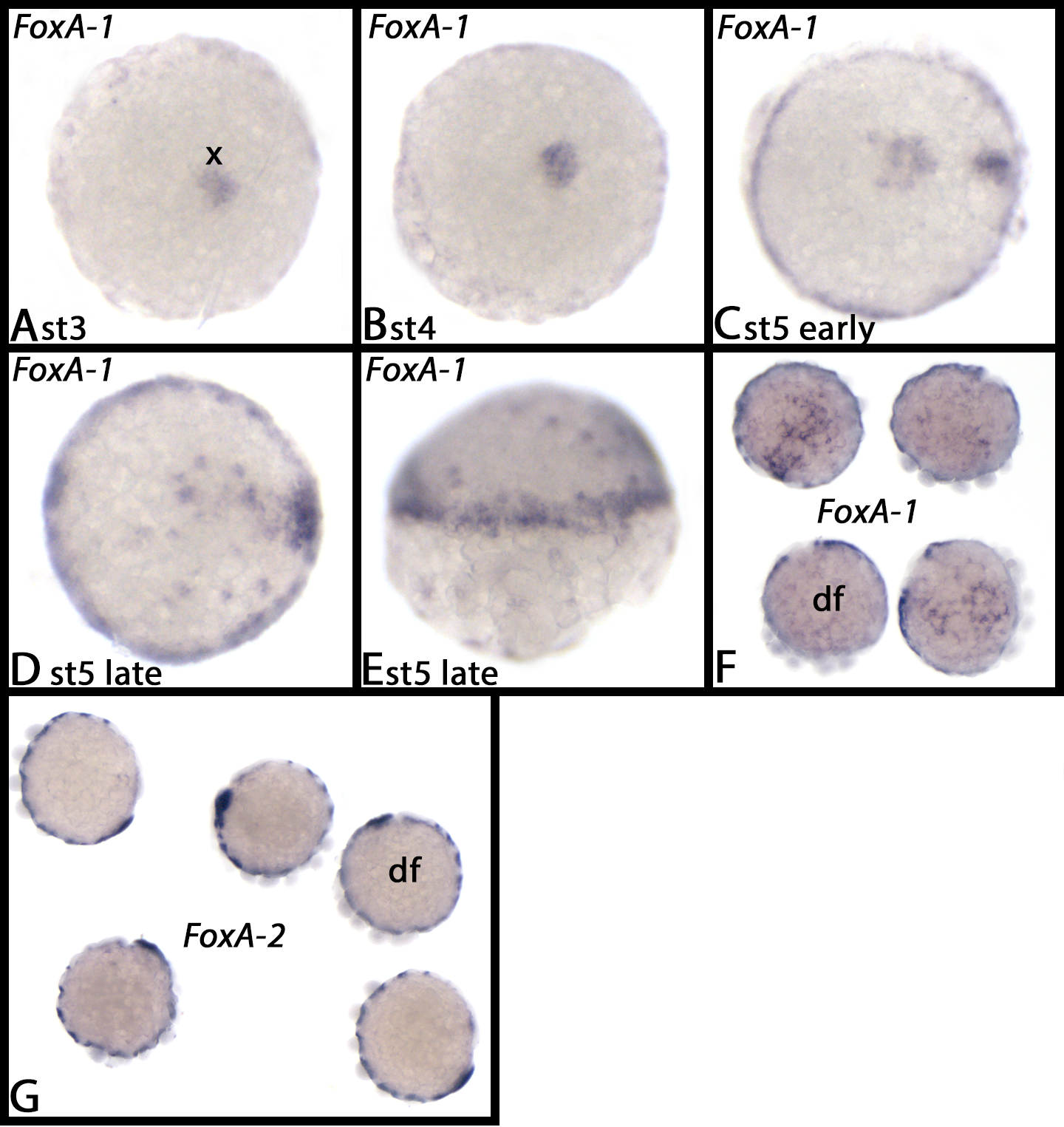

Supplement: S4 Fig — Early expression of FoxA-1 (A-F) and comparison of expression of FoxA-1 (F) and FoxA-2 (G) in the dorsal field. Note that FoxA-1, but not FoxA-2 is expressed in the dorsal field. The x in panel A marks the center of the germ disc that expresses FoxA1. Abbreviations in Table 2. (TIF) [file pone.0270790.s006.tif]

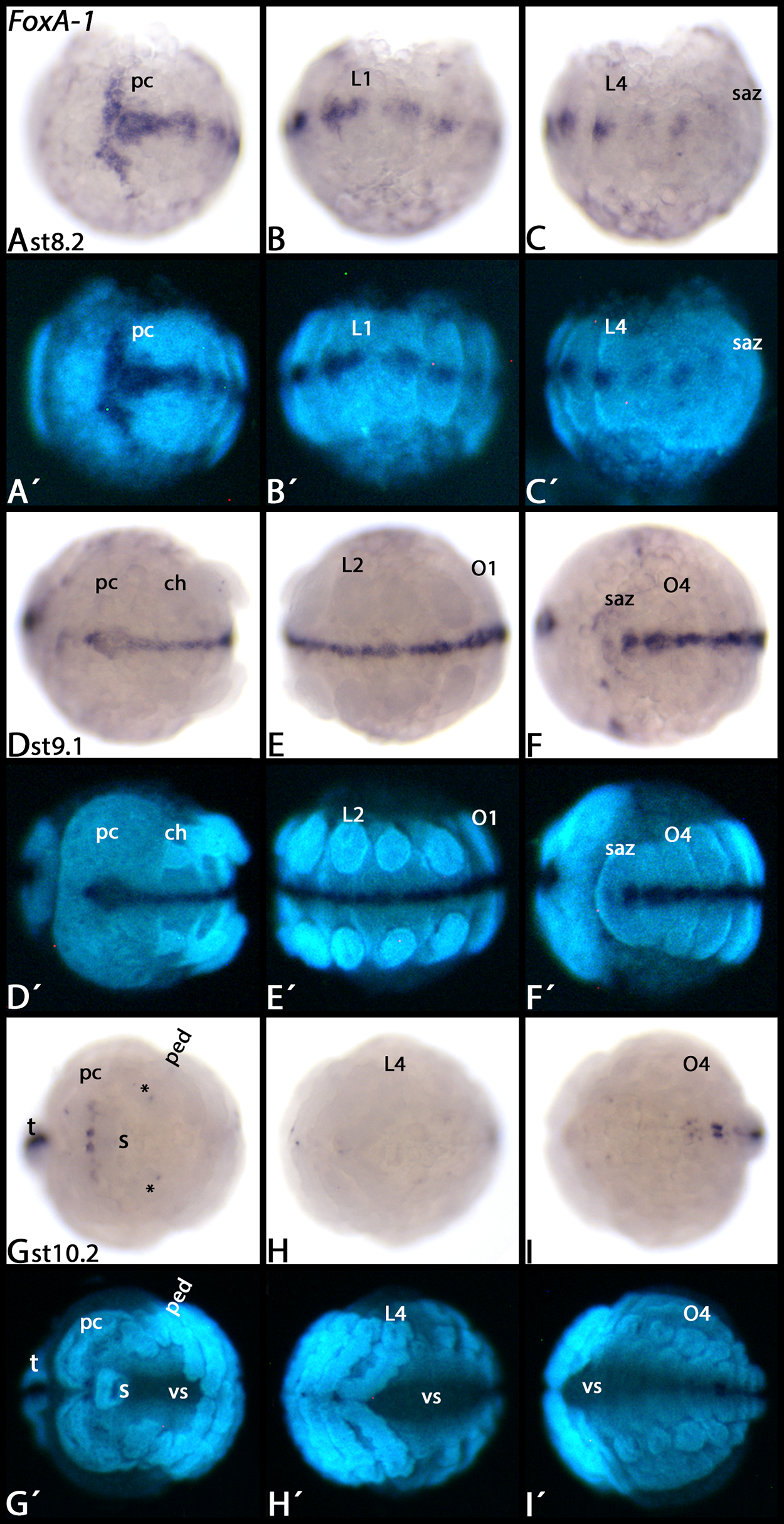

Supplement: S5 Fig — In all panels, anterior is to the left, ventral view. Panels A, D and G, view of anterior with head. Panels B, E and H view of middle part with walking limbs. Panels C, F, and I view of opisthosoma. Asterisks in panel G mark expression in the chelicerae. A´-I´ represent DAPI staining of the embryos shown in A-I. Each row (e.g. A-C) represents the same embryo. Abbreviations in Table 2. (TIF) [file pone.0270790.s007.tif]

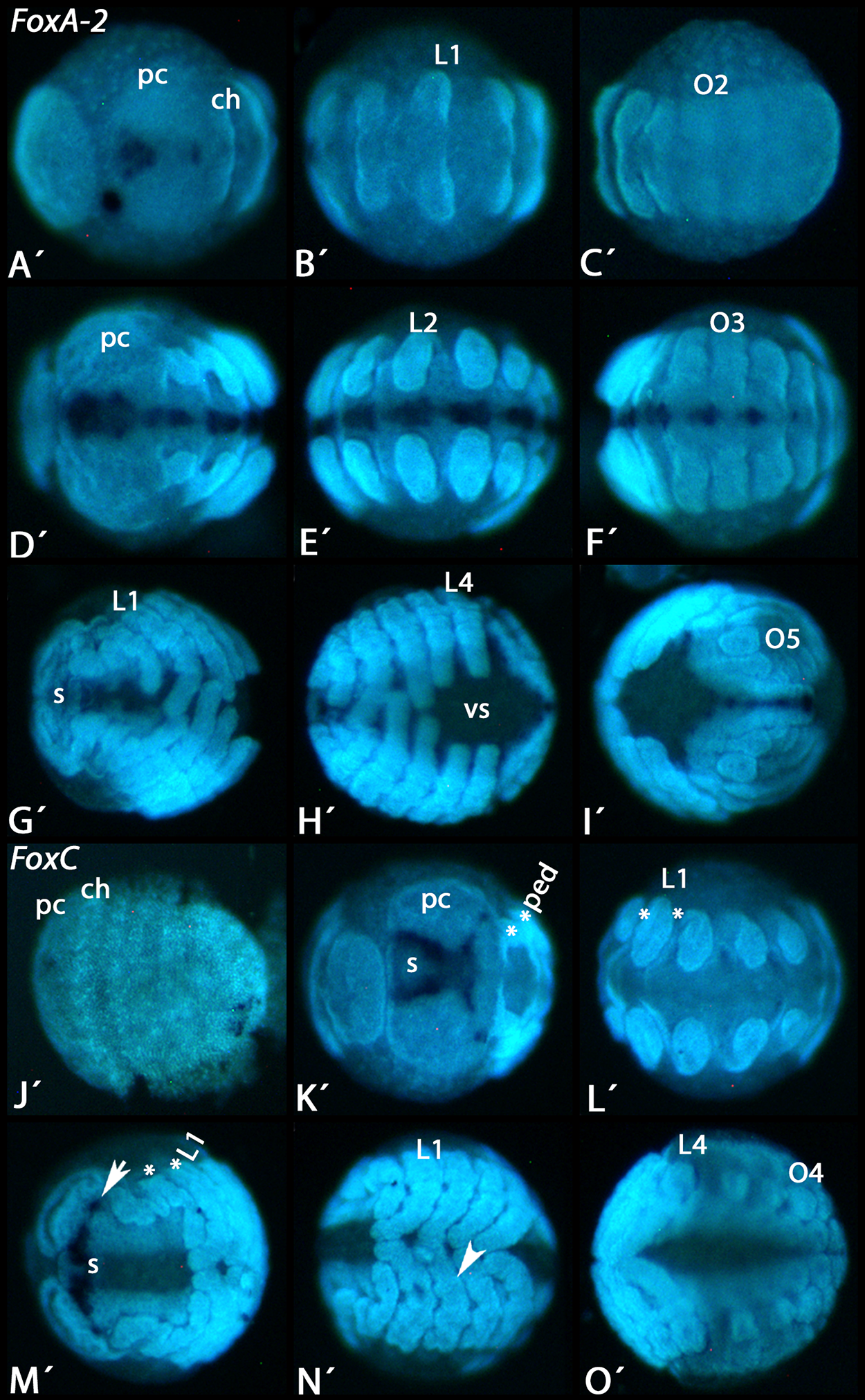

Supplement: S6 Fig — Abbreviations in Table 2. (TIF) [file pone.0270790.s008.tif]

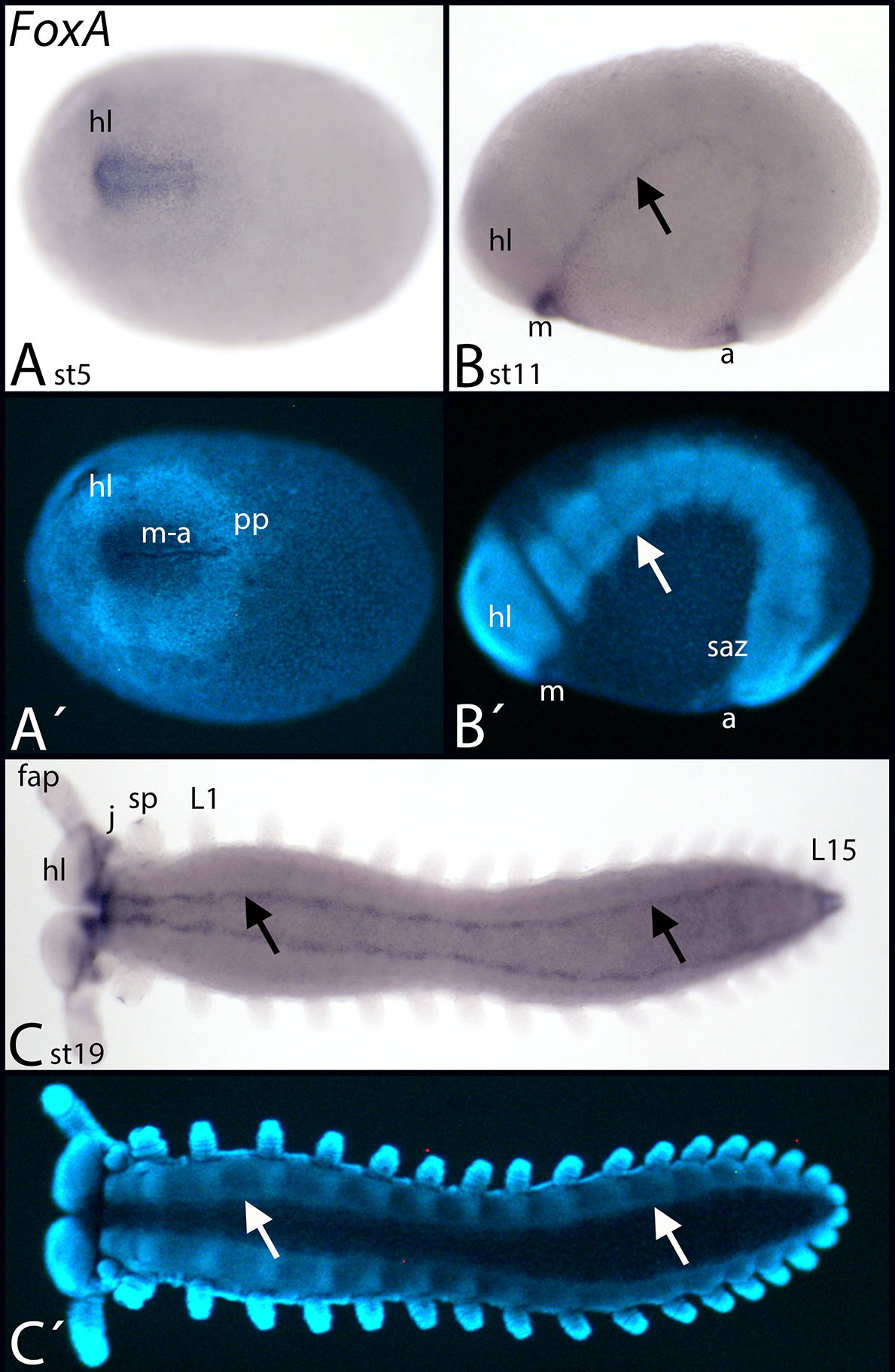

Supplement: S7 Fig — In all panels, anterior is to the left, ventral views, except panel B, lateral view, dorsal up. A´-C´ represent DAPI staining of the embryos shown in A-C. Arrows in panels B and C mark expression along the ventral margin of the embryo proper. Abbreviations in Table 2. (TIF) [file pone.0270790.s009.tif]

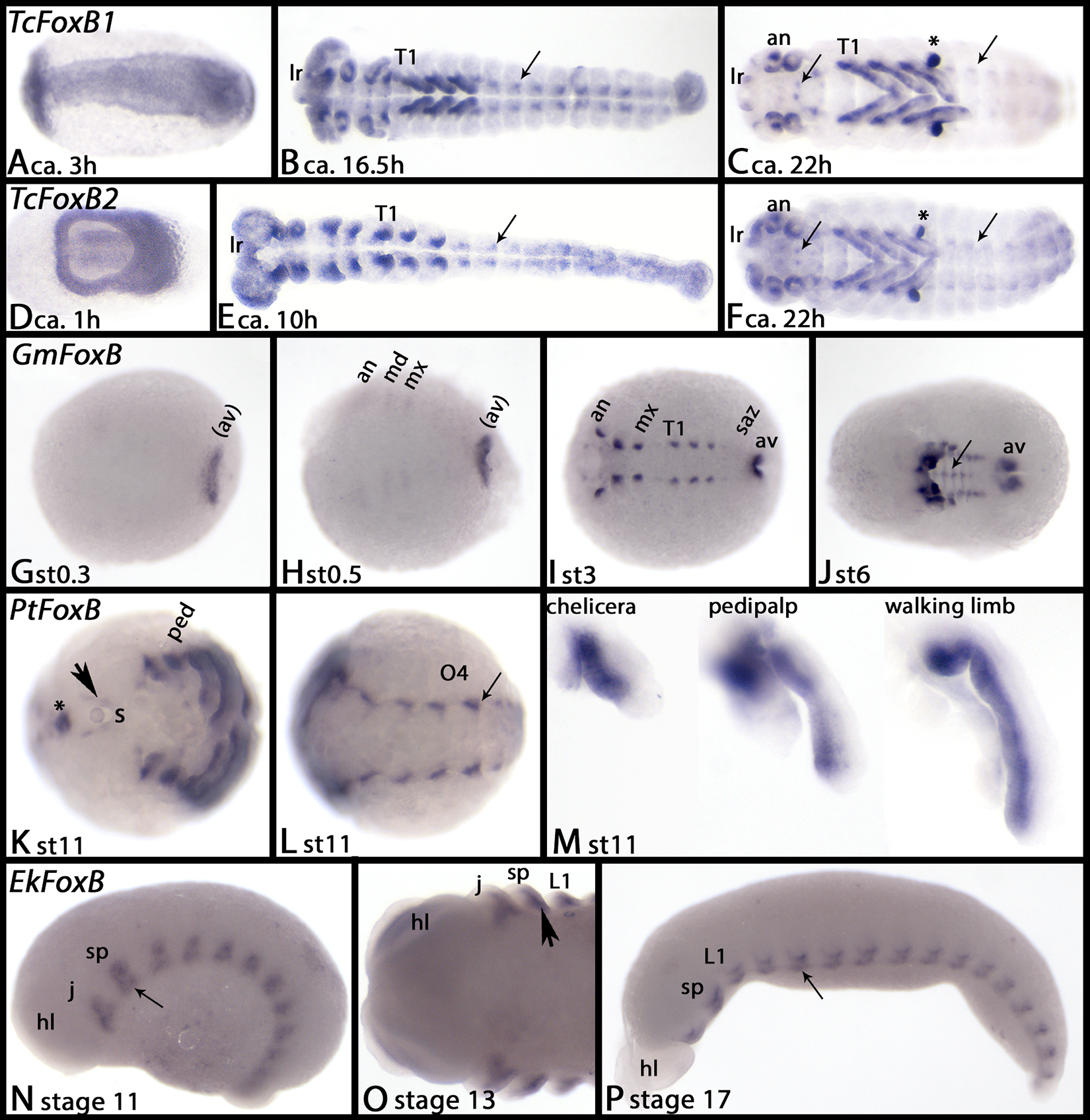

Supplement: S8 Fig — Expression of Tribolium FoxB1 and FoxB2 (A-F), Glomeris FoxB (G-J), Parasteatoda FoxB (K-M), and Euperipatoides FoxB (N-P). In all panels, anterior is to the left (except panel M, ventral to the left). All panels represent ventral views (except panels M, N and P, lateral views). Narrow arrows in panels C, E, F, L, N and P point to the ventral nervous system. The asterisks in panels C and F mark unspecific signal in the pleuropodia. The arrow in panel J points to the midline. The arrow in panel K points to expression around the mouth (stomodaeum). The asterisk in panel K marks expression in the posterior end of the embryo. The arrow in panel O points to expression in the ventral tissue of the appendage. Abbreviations in Table 2. (TIF) [file pone.0270790.s010.tif]

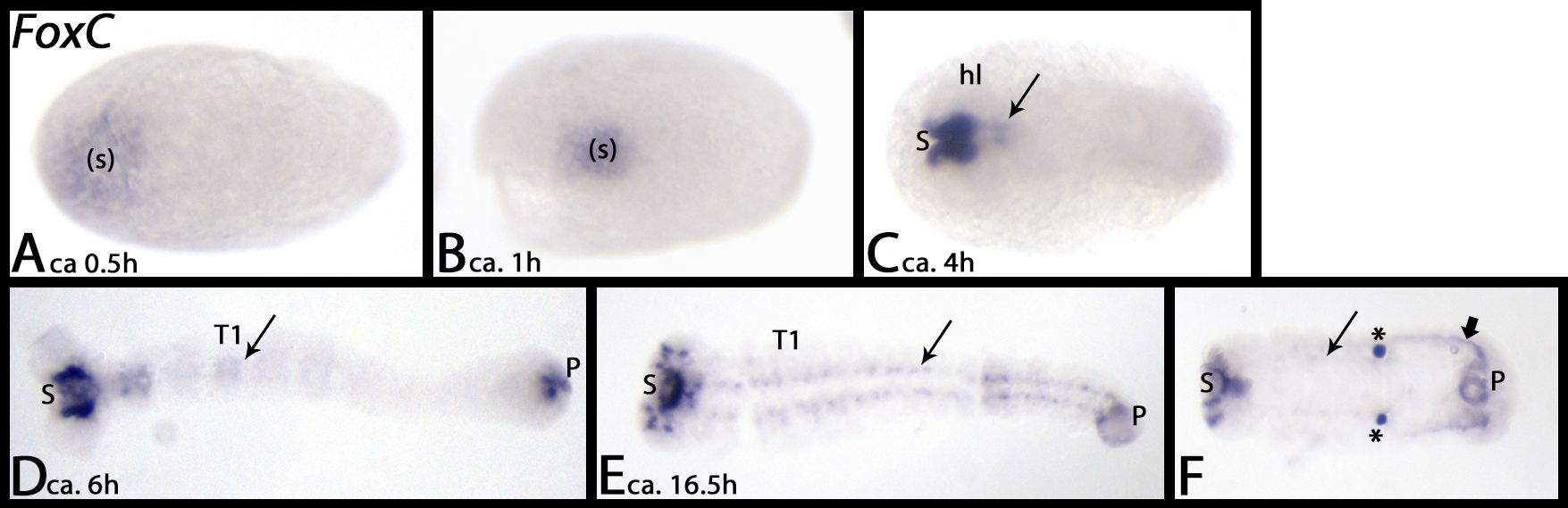

Supplement: S9 Fig — In all panels, anterior is to the left, ventral view. Embryos in D-F are flat-mounted. Long arrows in panels C-F mark expression in the VNS. Short arrow in F points to expression in dorsal tissue that could contribute to the heart. Asterisks in F mark unspecific staining in the pleuropodia. Abbreviations in Table 2. (TIF) [file pone.0270790.s011.tif]

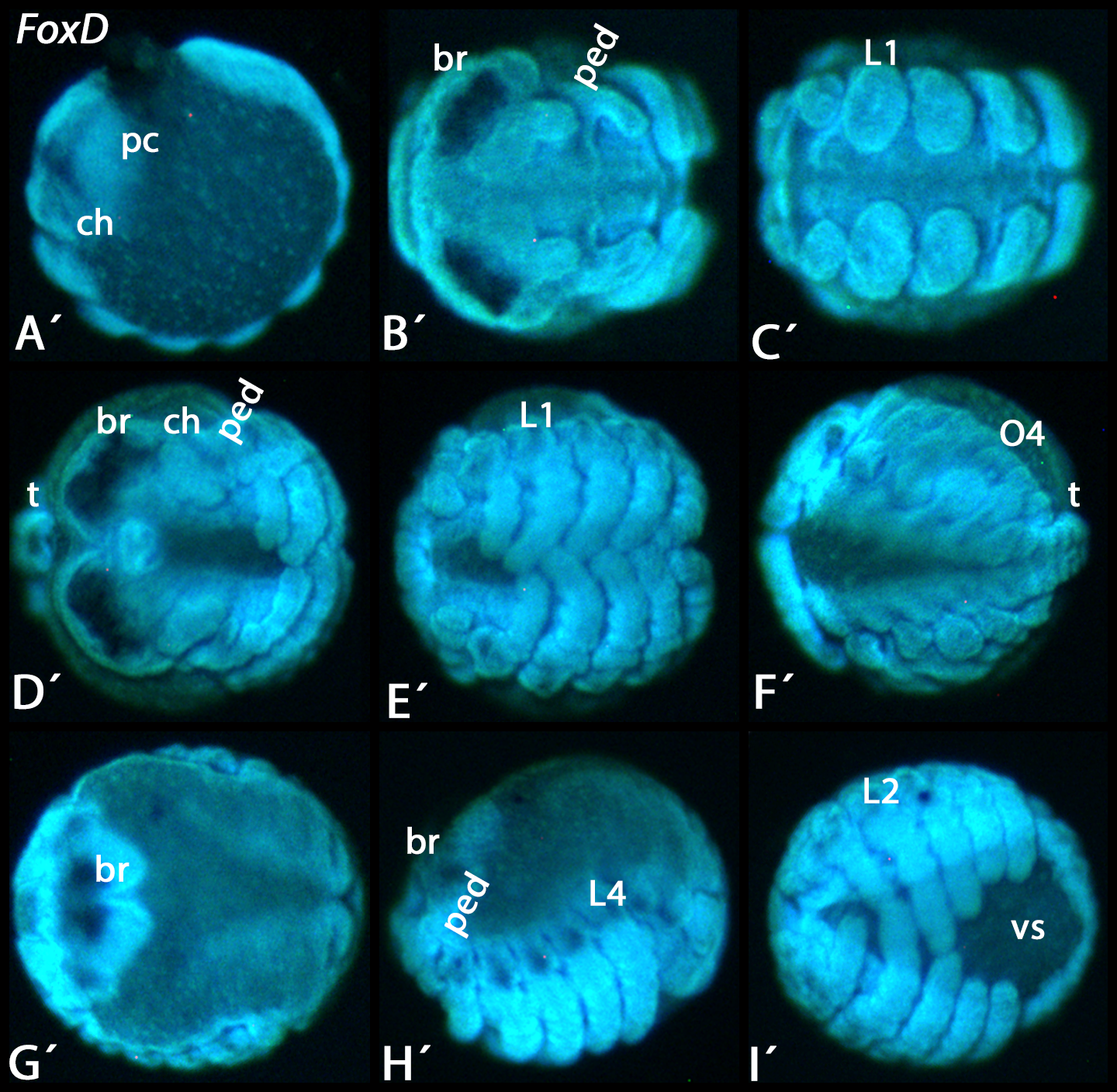

Supplement: S10 Fig — Abbreviations in Table 2. (TIF) [file pone.0270790.s012.tif]

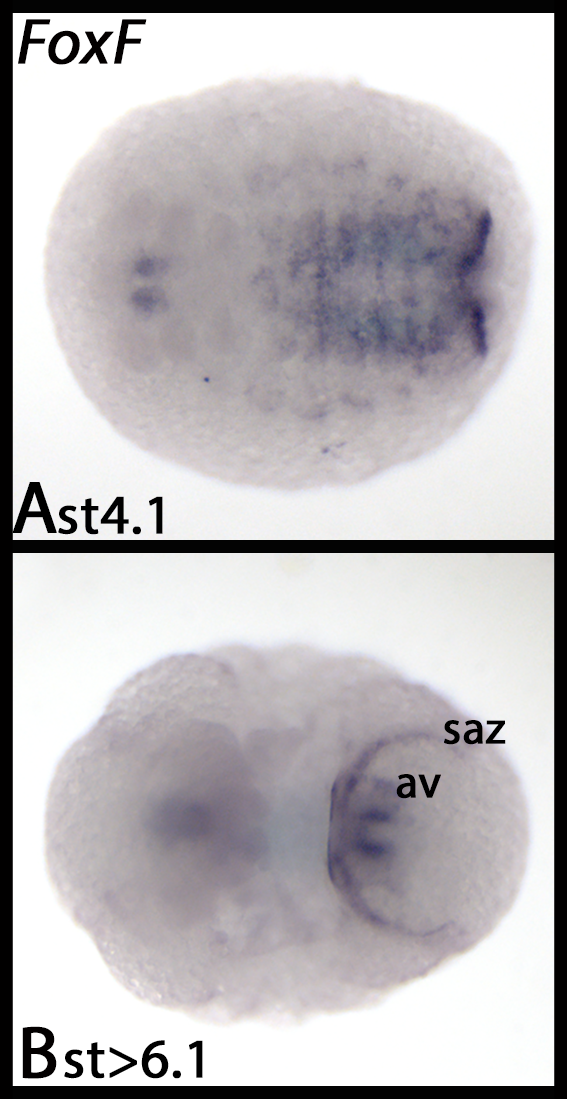

Supplement: S11 Fig — Anterior is to the left, ventral views. Abbreviations in Table 2. (TIF) [file pone.0270790.s013.tif]

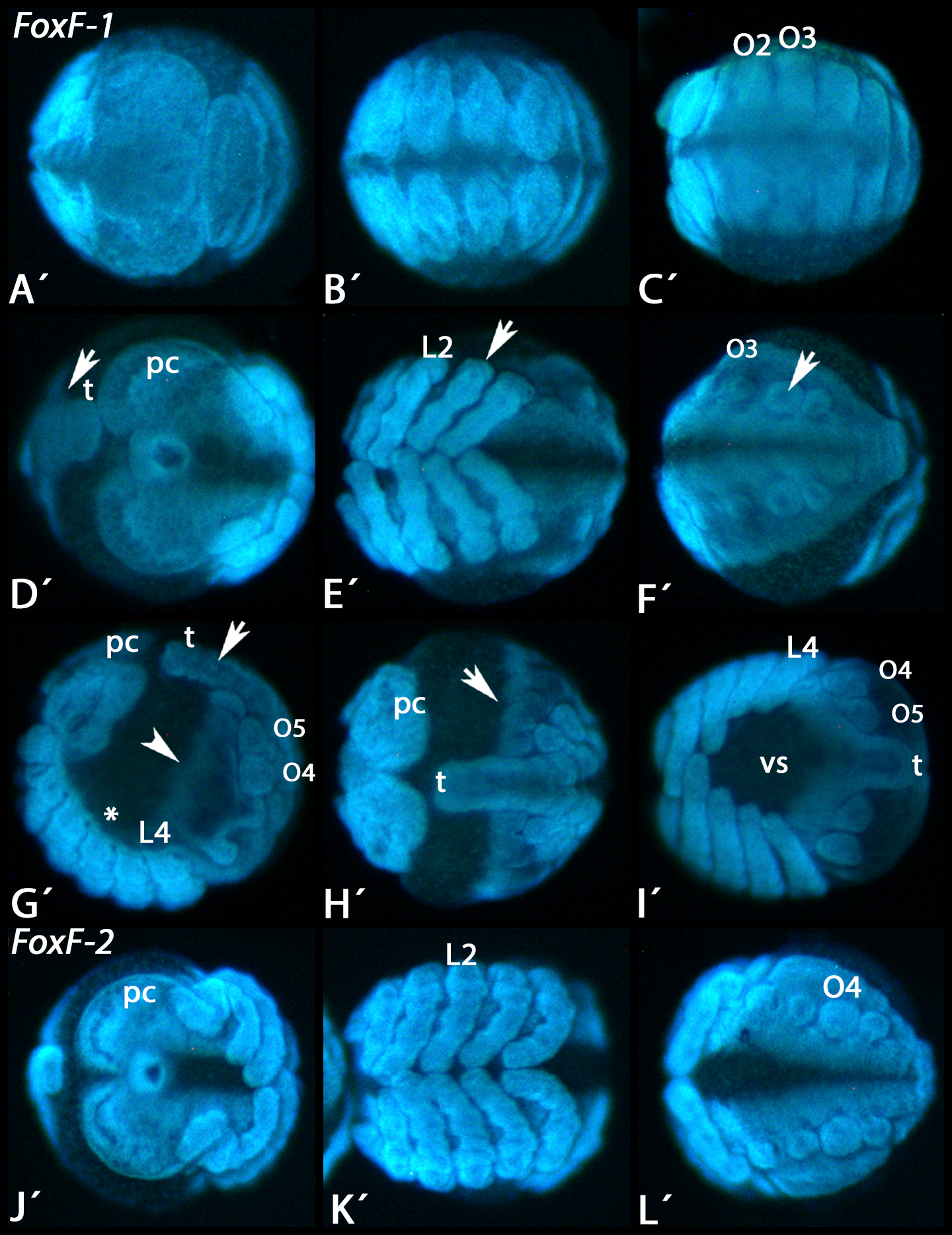

Supplement: S12 Fig — (TIF) [file pone.0270790.s014.tif]

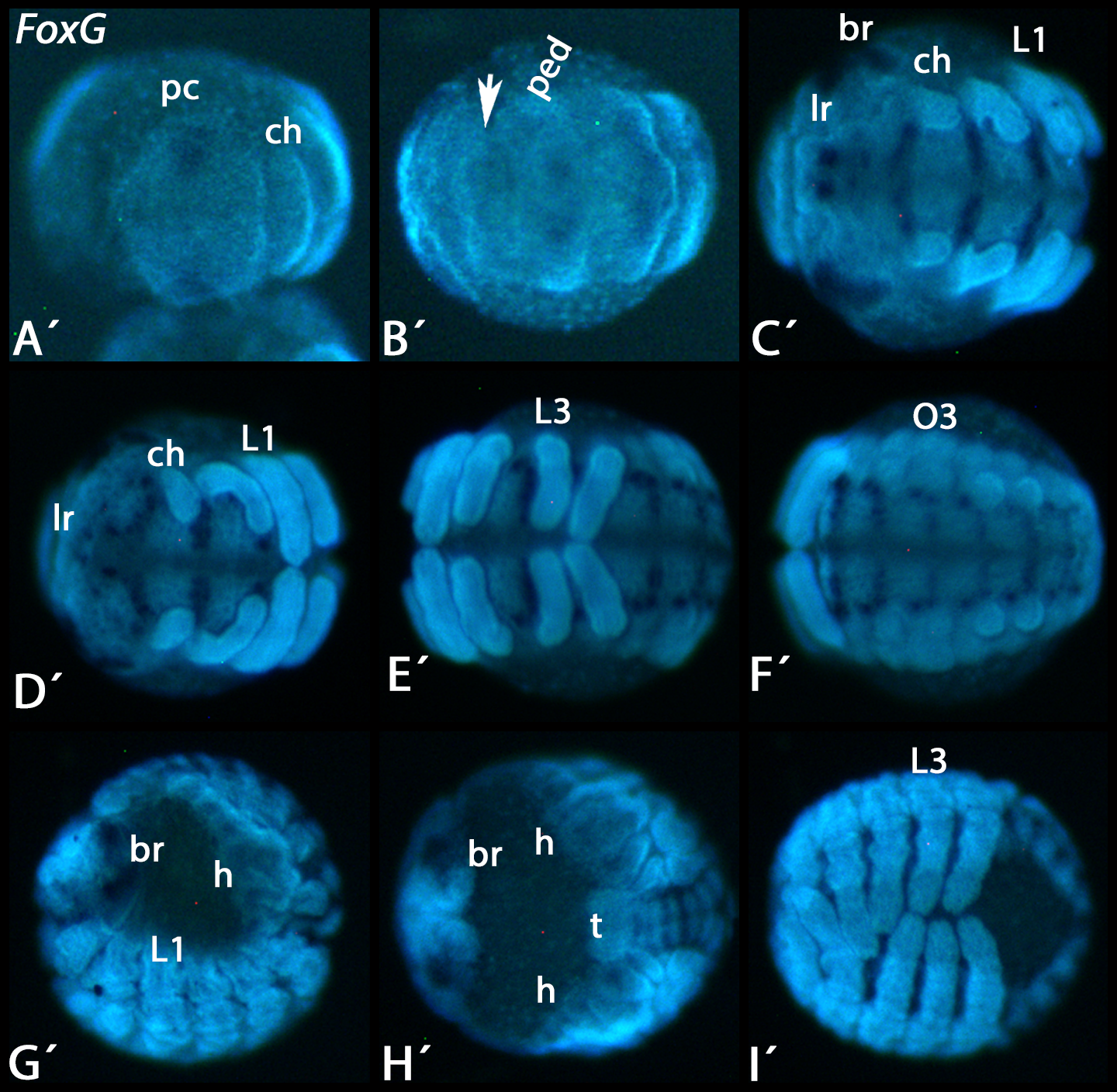

Supplement: S13 Fig — Abbreviations in Table 2. (TIF) [file pone.0270790.s015.tif]

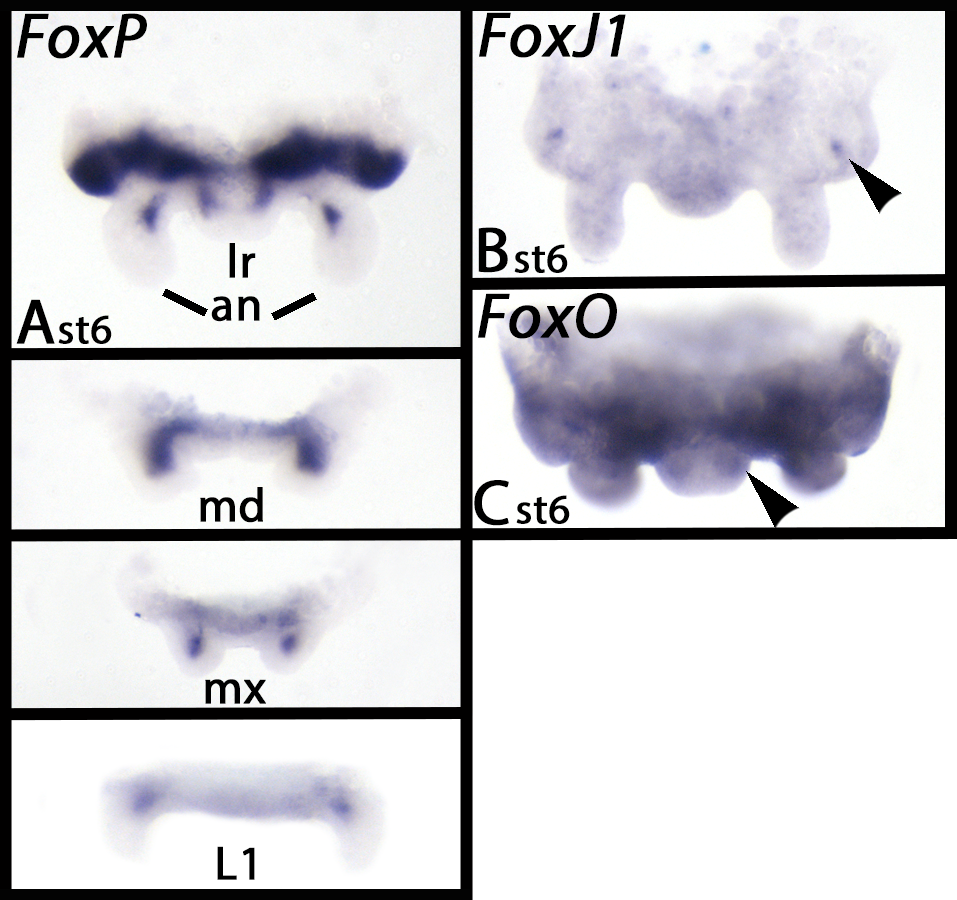

Supplement: S14 Fig — Expression of Glomeris FoxP (A), FoxJ1 (B) and FoxO (C), additional aspects. Anterior views. Arrowhead in panel B points to dot of expression in the lateral head. Arrowhead in panel C points to expression in the labrum. Abbreviations in Table 2. (TIF) [file pone.0270790.s016.tif]

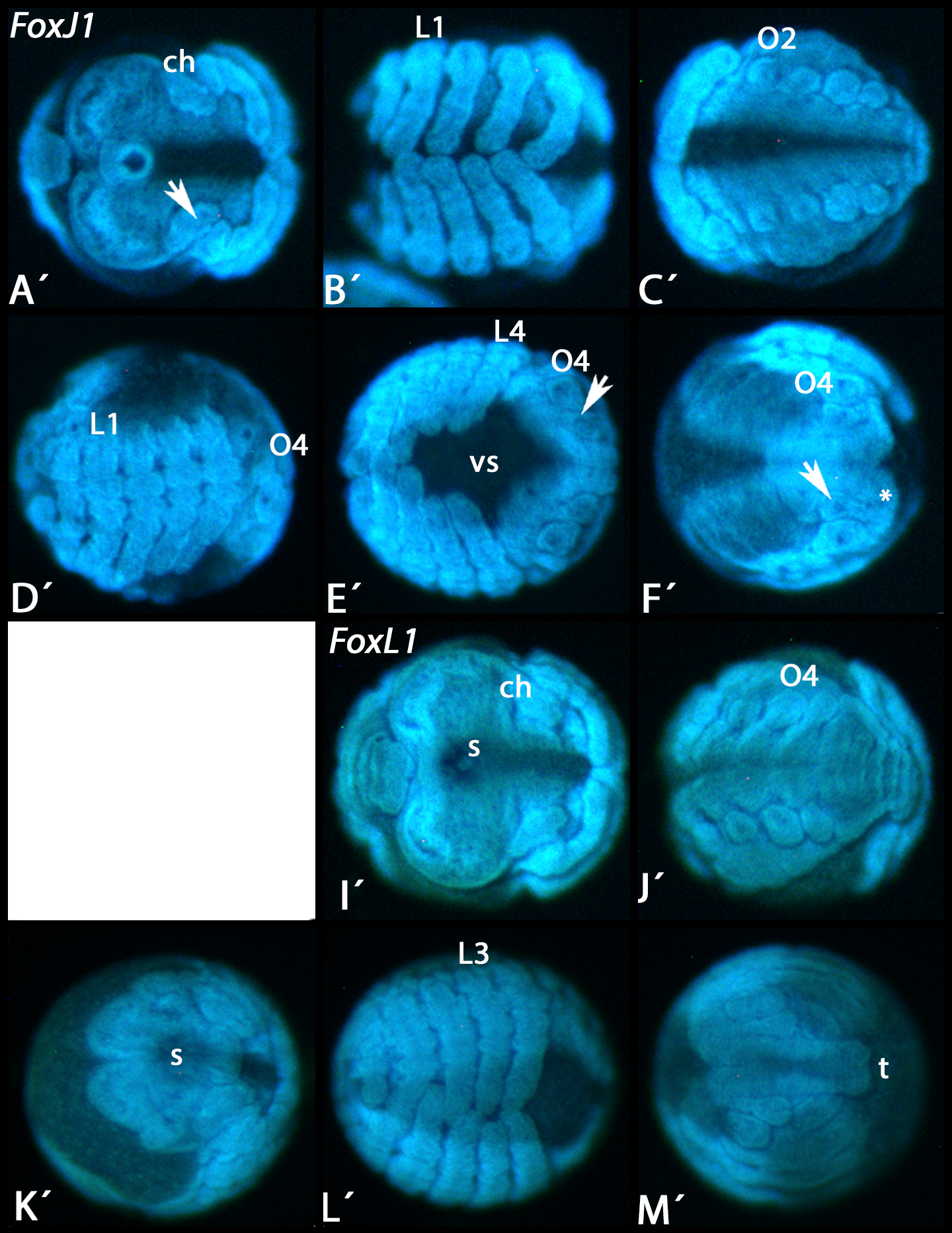

Supplement: S15 Fig — Abbreviations in Table 2. (TIF) [file pone.0270790.s017.tif]

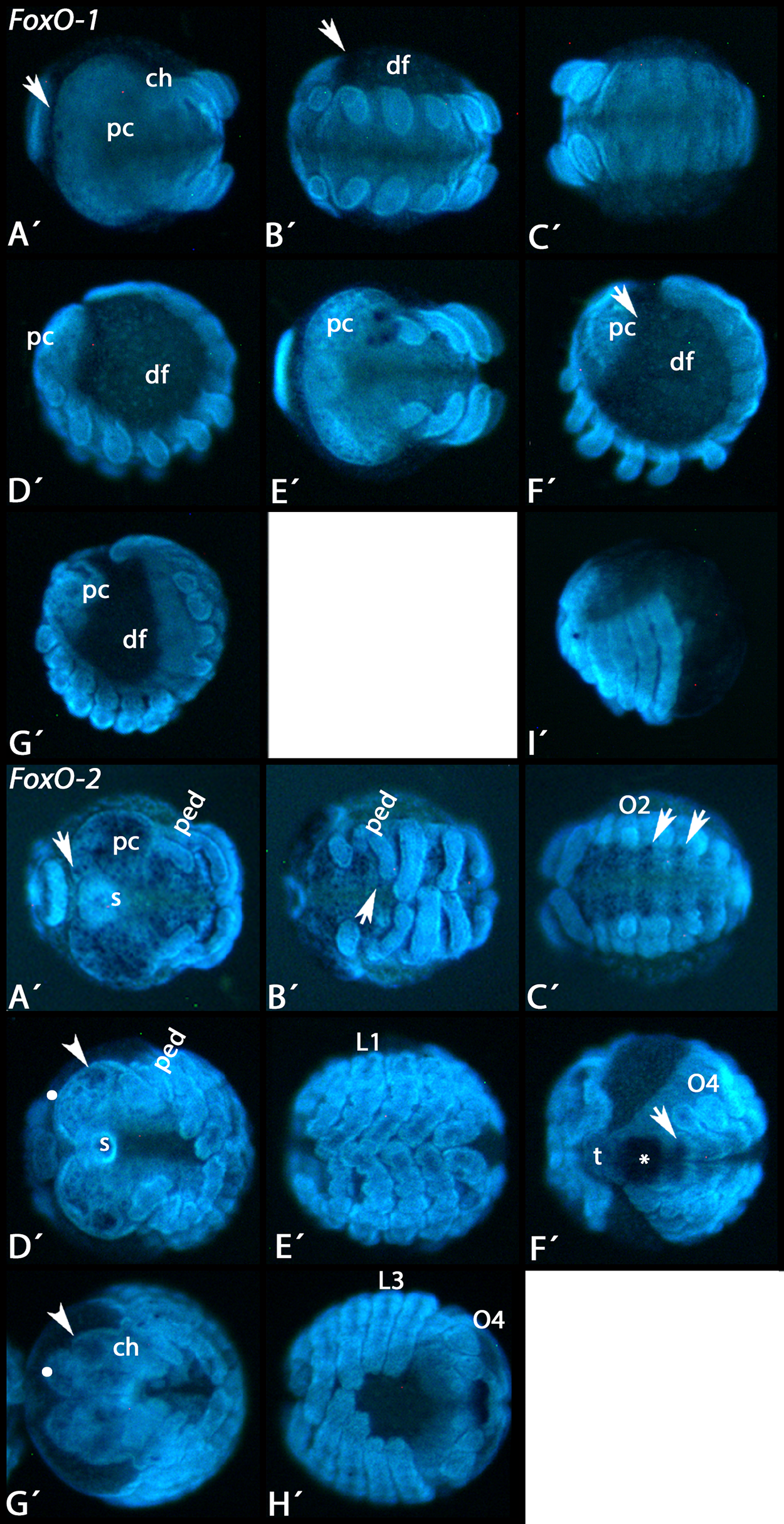

Supplement: S16 Fig — Abbreviations in Table 2. (TIF) [file pone.0270790.s018.tif]

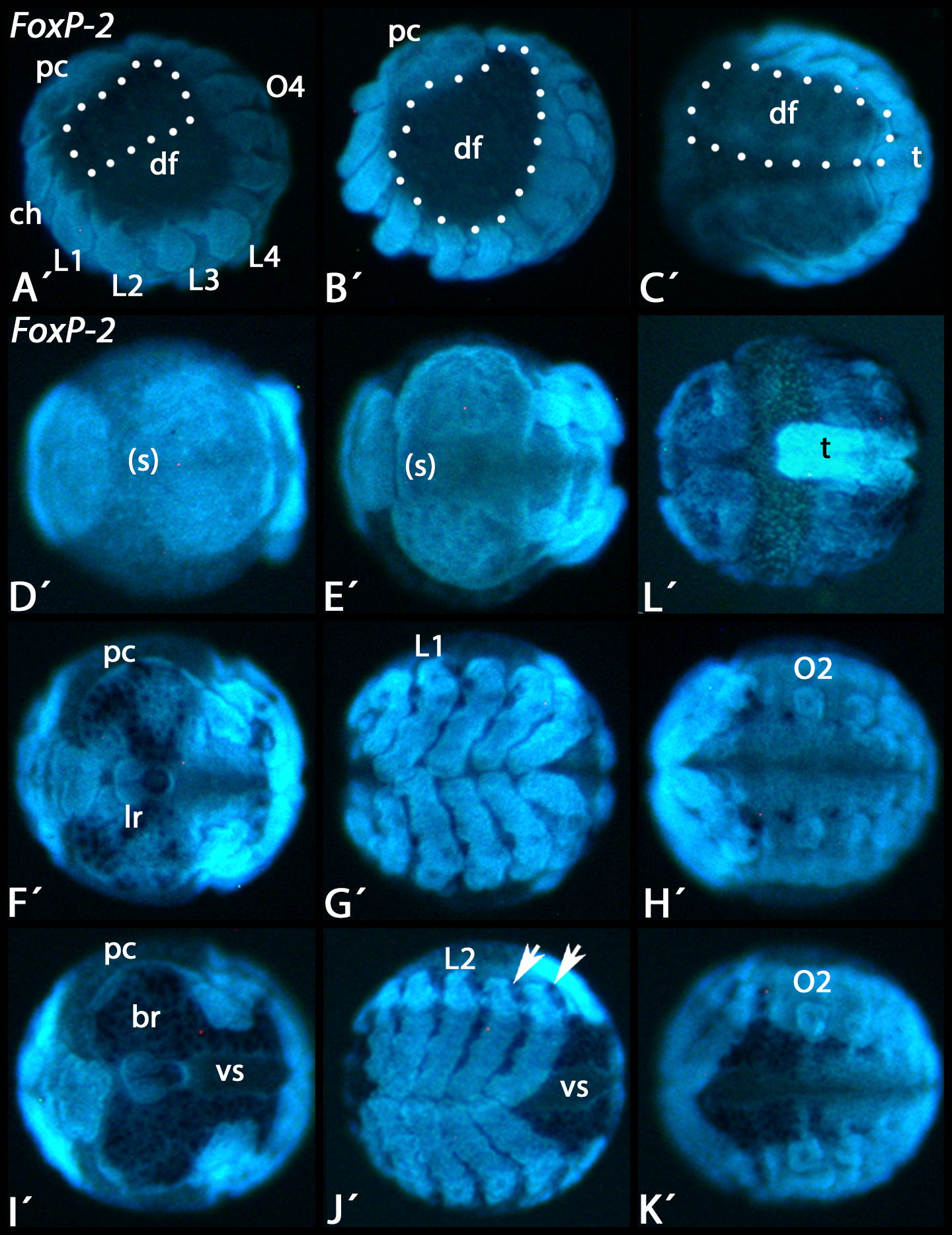

Supplement: S17 Fig — Abbreviations in Table 2. (TIF) [file pone.0270790.s019.tif]

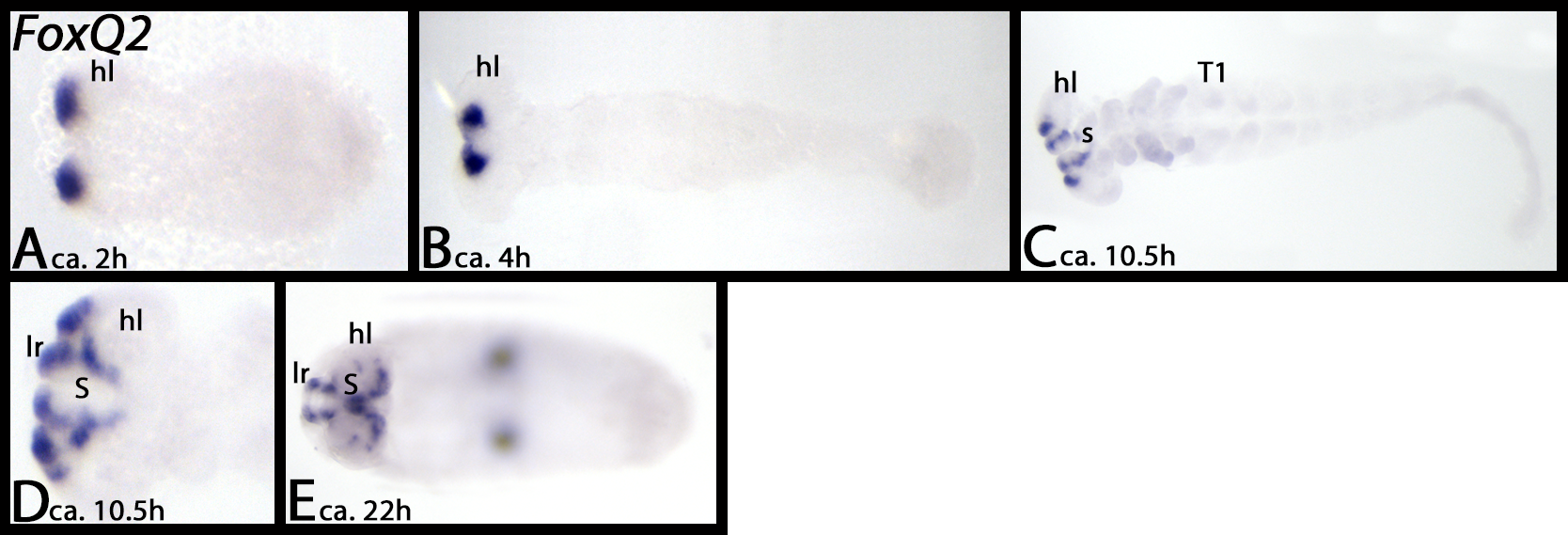

Supplement: S18 Fig — In all panels, anterior is to the left, ventral view. Embryos are flat-mounted, except embryo shown in panel A and E. The out-of-focus signal in the center of the embryo shown in panel E is in the pleuropodia that stain unspecific. Abbreviations in Table 2. (TIF) [file pone.0270790.s020.tif]

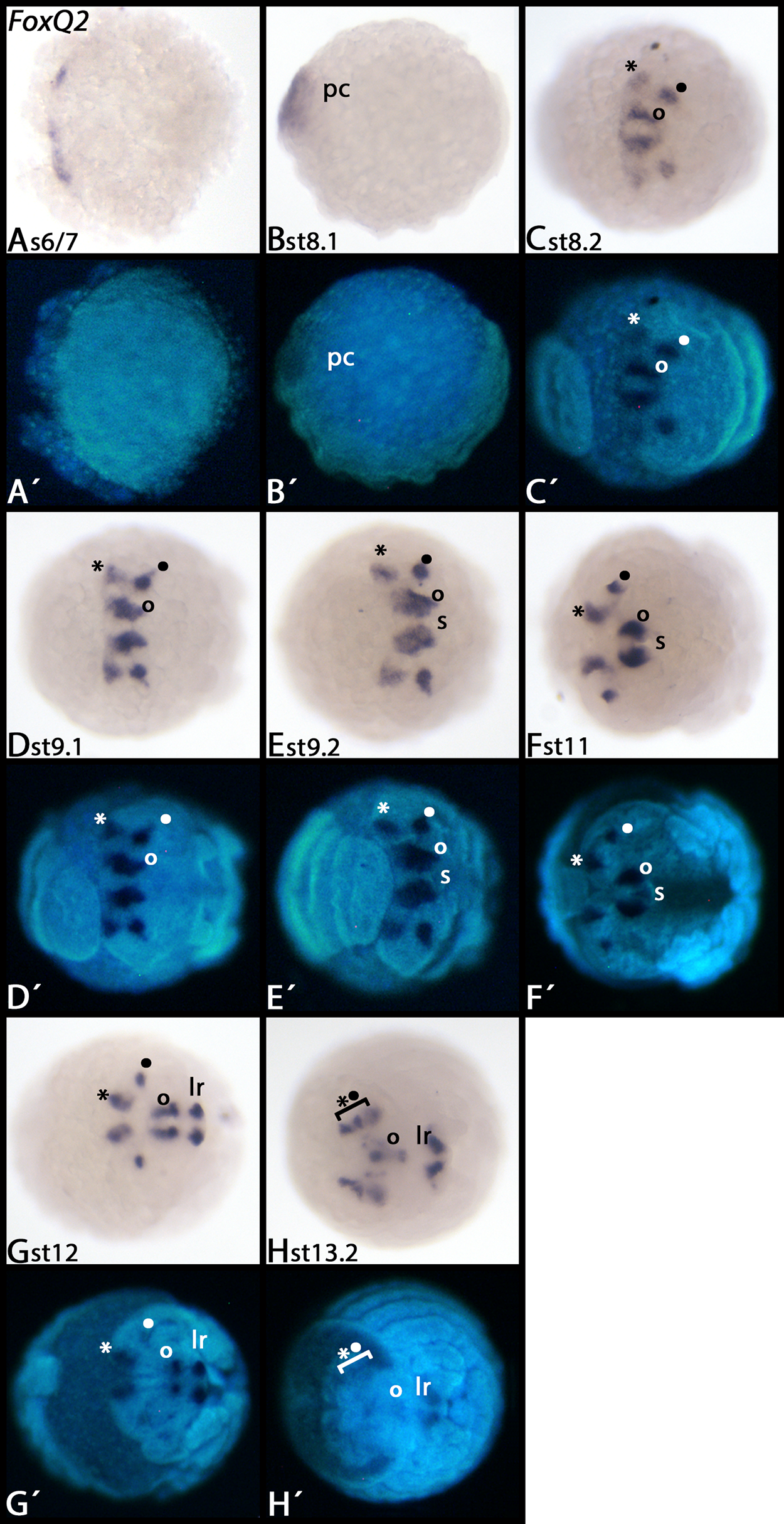

Supplement: S19 Fig — In all panels, anterior is to the left, anterior view, except panels B, lateral view. A´-H´ represent DAPI staining of the embryos shown in A-H. In all panels, asterisks, filled circles and open circles mark corresponding domains of expression during development. Abbreviations in Table 2. (TIF) [file pone.0270790.s021.tif]

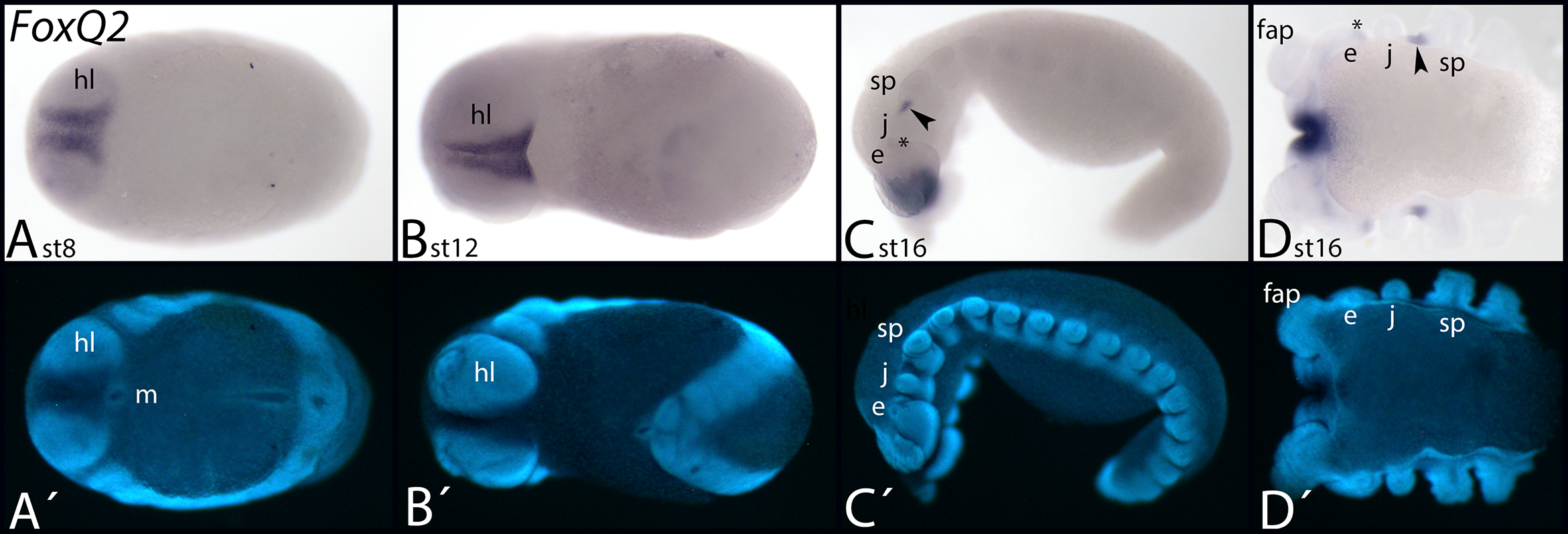

Supplement: S20 Fig — In all panels, anterior is to the left. Panels A and B, ventral view; panel C, lateral view, dorsal up; panel D, dorsal view. A´-D´ represent DAPI staining of the embryos shown in A-D. Asterisks in panels C and D mark faint expression ventral to the eyes. Arrowheads in panels C and D point to expression in the interface between jaws and slime papillae. Abbreviations in Table 2. (TIF) [file pone.0270790.s022.tif]
